# Calcium Sources Mitigate Salt Stress and Improve the Physiological and Productive Performance of Bell Peppers

**DOI:** 10.3390/plants15172620

**Published:** 2026-08-27

**Authors:** Daise Feitoza da Rocha, Enoch de Souza Ferreira, Antonia Gilvanira da Silva, Antonio Genilson Rodrigues Araújo, Gthielly Maíra Fernandes, Emanuel Araújo Alves, Francisco Felipe Barroso Pinto, Pedro Henrique de Araújo Gurgel, Francimar Maik da Silva Morais, Francisco de Assis de Oliveira, Nildo da Silva Dias, Miguel Ferreira Neto, Rafael Oliveira Batista, Hans Raj Gheyi, Alberto Soares de Melo, Antônio Gustavo de Luna Souto

**Affiliations:** 1 Department of Agricultural and Forestry Sciences, Federal Rural University of the Semi-Arid Region, Mossoró 59625-900, RN, Brazilgilvanira.silva92@gmail.com (A.G.d.S.); rafaelbatista@ufersa.edu.br (R.O.B.); 2 Academic Unit of Agricultural Engineering, Federal University of Campina Grande, Campina Grande 58429-140, PB, Brazil; hans.gheyi@ufcg.edu.br; 3 Department of Biology, State University of Paraíba, Campina Grande 58429-500, PB, Brazil; 4 Department of Crop Science, Federal University of Paraíba, Areia 58397-000, PB, Brazil

**Keywords:** *Capsicum annuum* L., complexed calcium, calcium nitrate, water salinity, physiology, production

## Abstract

Bell peppers (*Capsicum annuum* L.) are of great importance and have high productive potential in Brazil, especially in the Northeast region; however, their production is limited due to salinity problems in water and soil. The objective of this study was to evaluate sources and methods of calcium application for mitigating salt stress in bell pepper plants grown in a substrate-based hydroponic system. The experiment was conducted using a split-plot design in a 2 × 2 × 3 layout with four replicates, consisting of two application methods (substrate and spray), two ECiw levels (0.5 and 5.0 dS m^−1^), and three calcium sources (no calcium—NCa; calcium nitrate—Ca(NO_3_)_2_; calcium complexed with amino acids—Ca-AA). Forty-two days after transplanting, gas exchange analysis, chlorophyll a, b, and total chlorophyll indices, chlorophyll fluorescence, fruit number and weight, and yield per plant were measured. Ca-AA showed higher photosystem II efficiency when applied via the substrate and higher stomatal conductance (28.10%), resulted in higher fruit yield compared to the other treatments, and contributed to a reduction in apical rot. Meanwhile, the application of Ca(NO_3_)_2_ improved electron transport in photosystem II by 5.46%, gas exchange, and increased chlorophyll b and total chlorophyll indices by 29.62% and 10.93%, respectively. It is concluded that the application of Ca sources, especially Ca-AA, has the potential to mitigate the effects of salt stress on bell pepper plants in a substrate-based hydroponic system, improving their photosynthetic and productive performance.

## 1. Introduction

Native to Central America, the bell pepper (*Capsicum annuum* L.) belongs to the Solanaceae family and is one of the most economically important vegetables for global agriculture among the 40 species in the Solanaceae family [1]. According to EMBRAPA (Brazilian Agricultural Research Corporation), a large portion of Brazil’s bell pepper production comes from family farms with less than 5 hectares of land; in other words, in addition to supplying major commercial centers, this crop provides a livelihood for many families, even with limited access to technology [2]. Thus, bell peppers are a crop of great importance to Brazil.

Despite its significant economic importance, bell pepper cultivation faces serious constraints due to soil or water salinity, particularly in semi-arid regions, where water is limited in both quantity and quality [3]. In this region, surface or groundwater reservoirs often suffer from some degree of salinity in the water, yet they remain the only available source of water for crops [4], including bell peppers [5]. In terms of salinity tolerance, the crop is considered sensitive to irrigation with saline water, and its growth and yield are compromised in water with an electrical conductivity (EC) greater than 1.5 dS m^−1^ [6,7]. The use of this type of water, combined with a high rate of evapotranspiration, causes soluble salts to accumulate in the soil, making salt stress a reality for many bell pepper growers in this region [8].

Soil salinity impairs plant growth by reducing the soil’s osmotic potential, which promotes the accumulation of toxic ions in plant tissues and limits the root’s uptake of water and nutrients [9]. In plants, excess salts in the solution cause stomatal closure and reduce the plant’s ability to take up atmospheric CO_2_, thereby impairing photosynthesis, growth, and crop yield [10,11]. In addition, salinity affects electron transport processes, the stability of thylakoid membranes, and the photochemical efficiency of photosystem II (PSII) [12]; it also causes an ionic imbalance, reducing the uptake of calcium (Ca^2+^) by plants due to the accumulation of sodium (Na^+^) [13]. As a result, Ca deficiency in plants can impair the formation of cell wall components and the cellular signaling mechanisms involved in the stress response, increasing susceptibility to cellular damage, especially in developing fruits. Thus, this damage results in the disintegration of plasma and tonoplast membranes and causes rupture of the endoplasmic reticulum, culminating in necrosis in the fruits, known in bell peppers as apical rot, a nutritional disorder caused by low calcium supply to the plants [14]. This damage results in stunted crop growth, making it necessary to implement management strategies under saline conditions [4].

Given the negative impacts on crop growth and yield in recent decades, strategies involving mineral nutrition have been evaluated as a way to mitigate salt stress in plants. In this regard, the supply of calcium to plants has shown promise in mitigating salt stress [15]. The role of Ca^2+^ is associated with an increase in the selectivity of K^+^ over Na^+^ [16,17]. Furthermore, it acts as a stress signal, regulating vital metabolic processes in plants, such as the regulation of stomatal opening, the activation of antioxidant enzymes, and the accumulation of osmoprotectants that reduce the accumulation of reactive oxygen species (ROS), thereby contributing to the maintenance of the plant’s water status [15,18]. These mechanisms, combined with calcium’s role in maintaining the integrity of cell membranes, help reduce damage caused by salinity and support plant growth [19,20]. In addition, the exogenous application of calcium promotes the biosynthesis of chlorophylls while inhibiting their degradation, and improves the efficiency of electron transport in photosystem II [21].

Calcium fertilization has certain limitations due to the low mobility of this nutrient within plants. When applied through the soil, its uptake depends on pH, soil type, interactions with other nutrients, and soil and water salinity [22,23]. Due to these limitations, it is often recommended to apply calcium via spray application or through other technologies, such as chelation, to improve its uptake by plants [24]. Chelating agents, or complexing agents, are natural or synthetic compounds that bind to and surround minerals in order to facilitate their absorption by plants and prevent adverse interactions [25]. Calcium, for example, which has a positive charge, competes with other cations for entry into the roots and has difficulty being absorbed by the leaves, which have negative charges at their stomata; however, when chelated with amino acids, its physicochemical properties change, facilitating its absorption by the plant [26]. In this regard, the chelation of calcium with organic compounds, such as amino acids, can enhance the translocation of nutrients within the plant, making them even more effective in mitigating salt stress, since they are less polar than inorganic salts, exhibit greater mobility within the plant, and prevent undesirable reactions [26], which leads to greater absorption and translocation to organs with the greatest need, such as meristematic growth sites and fruits [27].

However, data on the application of efficient calcium sources, such as calcium chelated with amino acids, in pepper plants under salinity stress in semi-arid regions are scarce. Information of this nature will help reduce the damage caused by salt stress to the crop and serve as a basis for bell pepper production in areas with salinity problems. Based on the available information, the application of amino acid-complexed calcium, especially when applied via spray application, may reduce the effect of salt stress on the plants’ photosynthetic apparatus, promoting higher crop yields. In view of this, the objective of the present study was to evaluate the effects of calcium sources applied via soil and spray application on gas exchange, fluorescence, chlorophyll, and bell pepper production under saline stress in hydroponic cultivation conditions using a substrate.

## 2. Material and Methods

### 2.1. Location, Description of the Experimental Area, and Plant Material

**Figure 1:**
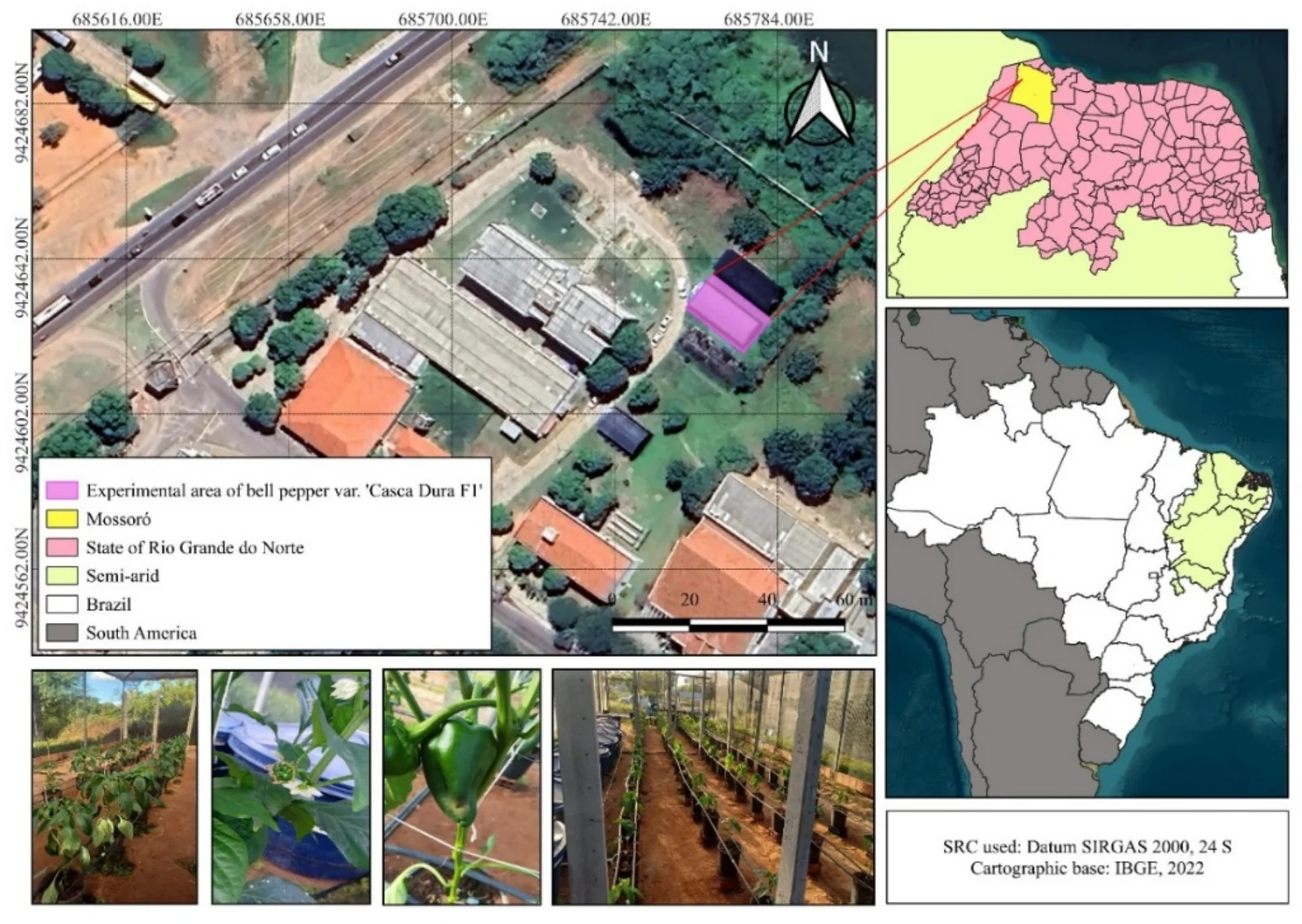
Map showing the location of the experimental area.

The experiment was conducted in a substrate-based hydroponic system from May to August 2025 in a greenhouse belonging to the Department of Forest Science and Agronomy at the Federal Rural University of the Semi-Arid Region (UFERSA), located in the municipality of Mossoró, Rio Grande do Norte State, Brazil (5°12′11″ S and 37°19′25.7″ W, at an elevation of 24 m above sea level), as shown in Figure 1. According to the Köppen climate classification, the region was characterized as semi-arid, type BSh—hot and dry—with an average annual precipitation of approximately 555 mm, an average air temperature of 28.4 °C, and an average relative humidity of around 63.25% [28,29].

**Figure 2:**
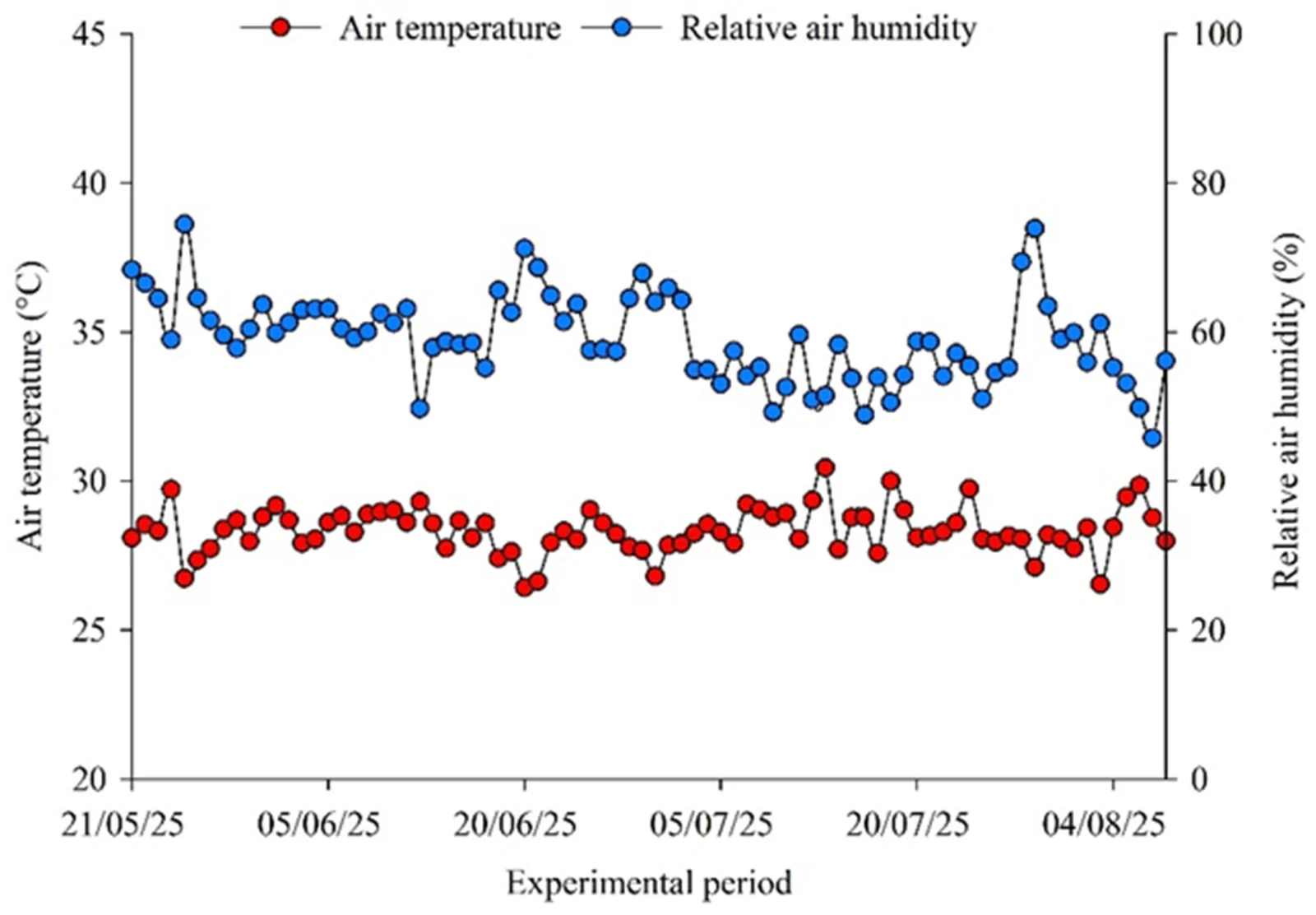
Air temperature and relative air humidity inside the greenhouse during the experimental period.

Throughout the experiment, air temperature and relative air humidity were continuously monitored using a digital thermo-hygrometer (HTC-2, KLX, Shenzhen, China). The continuous records were used to characterize the environmental conditions throughout the experimental period, as shown in Figure 2.

The plant material evaluated was bell pepper (*Capsicum annuum* L.) cv. Cascadura Ikeda, grown in 8 L pots containing, as substrate, a 2 cm layer of No. 2 gravel at the bottom of the pots and 6.5 L of pre-moistened and de-lumped coconut fiber, which provided good physical conditions for root growth and moisture management.

### 2.2. Treatment and Experimental Design

The experiment was conducted in a split-plot design, using a 2 × 2 × 3 layout, with four replicates. The main plot consisted of the two forms of calcium application methods—AM (substrate and spray). The subplots were composed of the two saline water—SW (0.5 and 5.0 dS m^−1^) and the three calcium sources—CaS (no calcium—NCa; calcium nitrate—Ca(NO_3_)_2_; calcium complexed with amino acids—Ca-AA). Each subplot was represented by a pot containing a bell pepper plant, which was considered the experimental unit. The control treatment consisted of plants irrigated with 0.5 dS m^−1^ water and without calcium.

### 2.3. Conducting the Experiment

Bell pepper seedlings (cv. Cascadura Ikeda) were supplied by Top Plant, a commercial company specialized in vegetable seedling production, located at Fazenda Agrícola Famosa, Icapuí, Ceará State, Brazil. Sowing was carried out on 30 April 2025, in 200-cell polyethylene trays filled with a commercial vegetable substrate based on shredded coconut husk fiber. The seedlings were transplanted into pots on 21 May 2025, that is, 21 days after sowing (DAS), when the seedlings exhibited adequate vigor and at least four true leaves, a condition considered ideal for establishment in a protected environment.

Following transplanting, water and nutrients were supplied via the irrigation system in accordance with the crop’s water and nutrient requirements throughout its phenological cycle, as recommended [30,31]. The low-salinity irrigation water (0.5 dS m^−1^) was sourced from the local water supply, and the saline water (5.0 dS m^−1^) was prepared by dissolving NaCl (P.A.) in low-salinity water (0.5 dS m^−1^), verifying the desired electrical conductivity (EC) with a portable conductivity meter (CDR-870, Instrutherm^®^, São Paulo, Brazil). Irrigation was conducted in nine daily pulses, each lasting 1 min and 44 s, with the number of pulses adjusted according to the phenological stages of the bell pepper, and the volume distributed throughout the experiment, as shown in Table 1.

The pulsing strategy was adopted to maintain greater consistency in the availability of water and nutrients in the coconut fiber substrate—an essential condition in substrate-based hydroponic systems—thereby reducing fluctuations in the pot solution and promoting experimental uniformity. To this end, the system was automated using a controller (“timer”) so that, with each activation (irrigation pulse), the plants would simultaneously receive water and nutrients, ensuring both water and nutrient replenishment.

In order to regulate the supply of water and nutrients to the plants, four 250 L tanks were installed inside the greenhouse. Two of them were used to store the stock solutions (A and B): solution A containing macronutrients and solution B containing micronutrients and the calcium source (Ca), since the Ca source could cause the precipitation of phosphorus (P) and sulfur (S) from the sources containing these nutrients. The remaining reservoirs were equipped with independent pumping systems, which were responsible for delivering irrigation water to the experimental plots. Each of these reservoirs was filled with water of different electrical conductivities (0.5 and 5.0 dS m^−1^). Whenever refilled, the tanks received nutrients from the stock solutions, ensuring uniformity in nutritional composition across the treatments, which differed only in the salinity level of the irrigation water. A standard nutrient solution with an electrical conductivity (EC) of 2.0 dS m^−1^, recommended for bell pepper cultivation, was used in all treatments. The stock solutions were prepared with non-saline water and injected into the irrigation tanks, which contained waters with EC values of 0.5 and 5.0 dS m^−1^. Consequently, the final fertigation solutions applied to the plants had EC values of 2.5 and 7.0 dS m^−1^, respectively. Thus, all treatments received the same nutrient composition, differing only in the salinity level of the irrigation solution.

**Figure 3:**
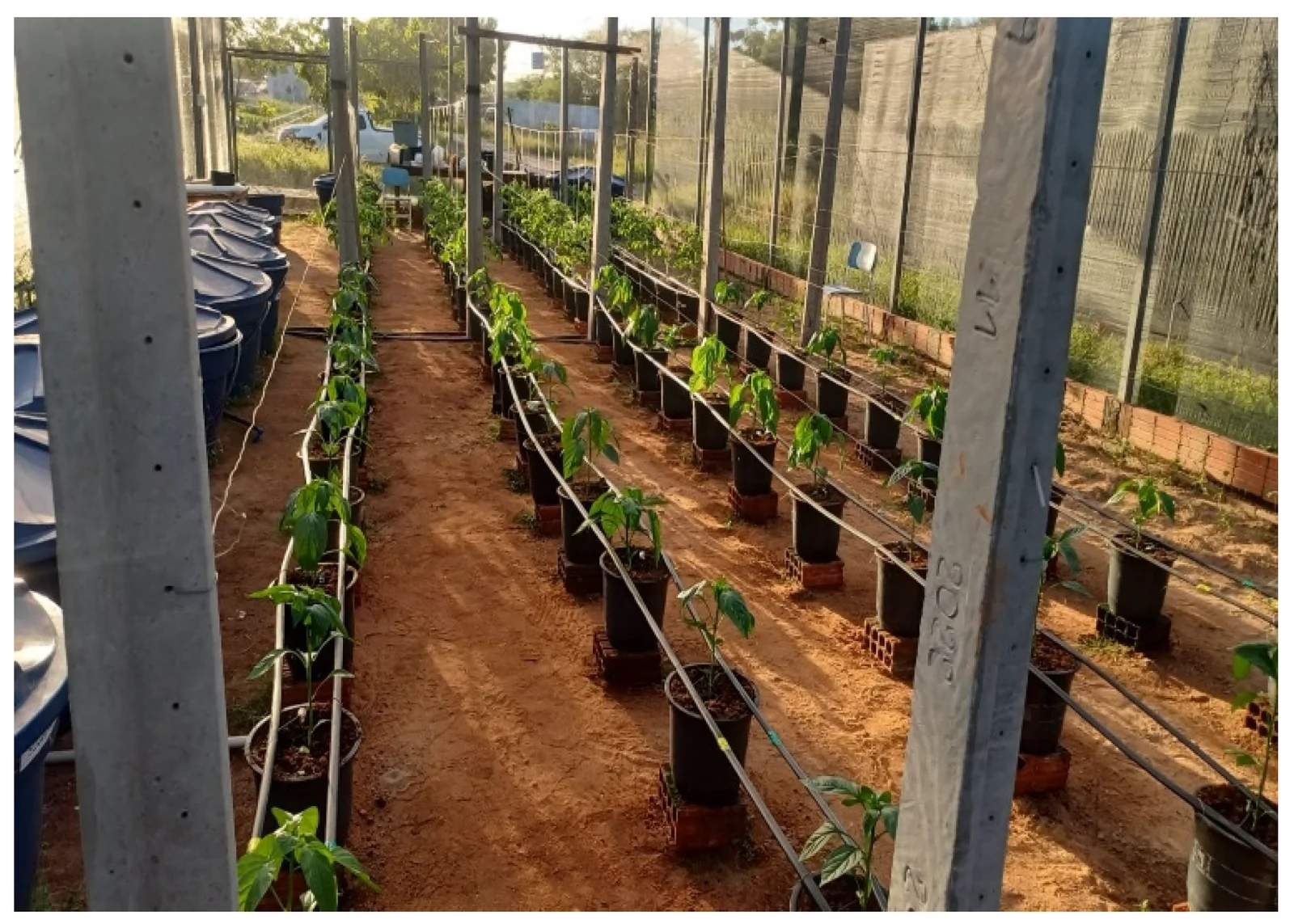
Drip irrigation system distributed across dual lines in each block.

The irrigation system used was a “drip” system, operating at a nominal flow rate of 5 mL s^−1^ per emitter (18 L h^−1^). For installation, 16 mm polyethylene pipes that were not perforated at the factory (referred to as “blind” pipes) were used, to which “drippers” were attached at the level of each pot, allowing for the targeted application of the nutrient solution to each experimental unit. To enable the delivery of the two water qualities (low and high salinity), double lines of tubing were installed in parallel, running through all experimental units as shown in Figure 3. This configuration ensured the precise and independent application of each water type with its respective electrical conductivity level, preventing cross-contamination between treatments and adhering to the randomization of the experimental design.

The nutrient solution used in the experiment was prepared according to the recommendations of [30] for bell pepper crops. The fertilizers used in the macronutrient formulation were: calcium nitrate (15.5% N and 19% Ca), potassium nitrate (12% N and 44% K_2_O), potassium sulfate (15% S and 48% K_2_O), monopotassium phosphate (52% P_2_O_5_ and 34% K_2_O), potassium chloride (60% K_2_O), magnesium sulfate (9% Mg and 12% S), and magnesium nitrate (11% N and 9.6% Mg), as well as the micronutrient complexes Dripsol Micro Rexene Fe EDDHA (6% Fe-EDDHA) and Dripsol Micro Equilíbrio (1.1% Mg, 0.85% B, 0.5% Cu-EDTA, 3.4% Fe-EDTA, 3.2% Mn-EDTA, 0.05% Mo, 4.2% Zn). To prepare the stock solutions, the following composition was used: Solution A, consisting of calcium nitrate (650 g per 1000 L), magnesium nitrate (50 g per 1000 L), and the micronutrient complexes (30 g per 1000 L), and Solution B, containing potassium nitrate (506 g per 1000 L), potassium phosphate (170 g per 1000 L), and magnesium sulfate (246 g per 1000 L). For the micronutrients, the commercial product Dripsol Micro Equilíbrio was used at the recommended dosage (30 g per 1000 L).

### 2.4. Sources of Calcium and Methods of Application

Calcium treatments were applied using calcium nitrate and calcium complexed with amino acids (Hendosar^®^). According to the manufacturer’s declared composition, Hendosar^®^ contains 7.15% Ca, 9.00% N, 6.00% K_2_O, 1.20% Mg, and the following amino acids: L-threonine (3.56%), L-aspartic acid (3.45%), L-serine (4.49%), L-glutamic acid (9.12%), L-proline (3.50%), L-glycine (2.43%), L-alanine (2.20%), L-cystine (2.45%), L-valine (3.10%), L-methionine (0.23%), L-isoleucine (1.70%), L-leucine (2.80%), L-tyrosine (1.02%), L-phenylalanine (1.78%), L-lysine (2.30%), L-histidine (0.90%), and L-arginine (5.20%). It was applied according to the experimental factor corresponding to the application method: spray or substrate (drench). Applications began seven DAT and were repeated every two weeks throughout the growing cycle, for a total of four applications, according to the methodology adapted by [31] for bell peppers grown hydroponically in substrate.

For spray application, a dosage of 5 g L^−1^ of (CaNO_3_)_2_ was used for Ca-AA, which is equivalent to a dosage of 8.6 mL L^−1^ (0.1094 g mL^−1^ of Ca). The dosages of the calcium sources were standardized according to the percentage and concentration of calcium, regardless of other nutrients or compounds in each source. Foliar spraying was performed manually, using a shield positioned over the rim of the pots to cover the entire surface area of the pot and the sides of the plants, with the aim of preventing drift and contact of the product with the growing medium and adjacent plants, thereby avoiding contamination and the effects of source factors and application methods.

Via the substrate, calcium nitrate was applied at a dosage of 0.19 g mL^−1^, and the amino acid-complexed calcium at a dosage of 0.1094 g mL^−1^ of Ca. The calcium dosages applied via the substrate were doubled due to the low mobility of the element in plants, since its transport occurs predominantly through the transpiration stream, which may lead to losses and competition with other ions in the solution [32].

### 2.5. Crop Management

During the experimental period, all necessary cultural practices, including staking, weeding, and phytosanitary management, were carried out to ensure adequate plant growth and health. Plants were staked using twine attached to horizontally installed support wires. Phytosanitary control was performed uniformly across all experimental units as a preventive and curative management strategy whenever pest or disease occurrence required intervention. Insecticides based on imidacloprid + beta-cyfluthrin (Connect^®^), acetamiprid (Rikolto^®^ and Acetamiprid^®^), abamectin (Batent^®^), and imidacloprid (Provado^®^) were applied according to the manufacturers’ recommendations. The main pests observed during the experiment were whiteflies (*Bemisia tabaci*), grasshoppers (*Schistocerca* spp.), mealybugs (*Phenacoccus* sp.), and leafminer larvae (*Liriomyza huidobrensis*).

### 2.6. Variables Analyzed

At 42 DAT, when the plants were in full bloom, gas exchange analysis was conducted between 8:00 and 11:00 a.m., using a GFS 3000FL infrared gas analyzer (IRGA) (Walz, Effeltrich, Germany), with artificial light at a photon density of 1200 µmol m^−2^ s^−1^ and a CO_2_ concentration of 400 ppm, using the youngest, fully expanded leaf as the index leaf [31]. The gas exchange variables analyzed were: CO_2_ assimilation rate (A, μmol CO_2_ m^−2^ s^−1^), stomatal conductance (gs, mol H_2_O m^−2^ s^−1^), internal CO_2_ concentration (Ci, µmol CO_2_ m^−2^ s^−1^), leaf transpiration (E, mmol H_2_O m^−2^ s^−1^), water use efficiency (WUE, μmol CO_2_ m^−2^ s^−1^) (mmol H_2_O m^−2^ s^−1^)^−1^, intrinsic water use efficiency (iWUE, μmol CO_2_ m^−2^ s^−1^) (mol H_2_O m^−2^ s^−1^)^−1^ and instantaneous carboxylation efficiency (iCE, (μmol CO_2_ m^−2^ s^−1^) (μmol CO_2_ m^−2^ s^−1^)^−1^. In addition, on the same gas exchange leaves, leaf chlorophyll indices (chlorophyll a index—Chl*a*, chlorophyll b index—Chl*b*, and total chlorophyll index—*T*Chl) were determined, with measurements taken at the basal, mid, and apical parts of the leaves, using a portable chlorophyll meter (ClorofiLOG^®^ CFL 2060, Falker, Porto Alegre, Brazil). Based on these values, the mean value of the variables was determined.

During the same period, chlorophyll fluorescence was measured at night (between 7:00 and 9:00 p.m.) after at least 20 min of dark adaptation to ensure complete opening of PSII reaction centers and relaxation of most non-photochemical quenching (NPQ). Minimum fluorescence (Fo) and maximum fluorescence (Fm) were measured using a portable fluorometer (OS-30p+, Opti-Sciences Inc., Hudson, NH, USA). The fluorometer automatically calculated and displayed variable fluorescence (Fv = Fm − Fo), the maximum quantum efficiency of PSII (Fv/Fm). Minimum fluorescence (Fo) was recorded using a low-intensity modulated measuring beam, followed by a saturating light pulse to determine maximum fluorescence (Fm), following the procedures described by [33].

Harvesting began at 54 DAT and ended at 75 DAT; three times a week, the number of fruits (NF) was counted and fruit mass (MF) was determined using a semi-analytical scale (precision = 0.001 g). Fruit yield per plant (PRO) was calculated as the product of MF and NF. Bell peppers were considered ready for harvest when they exhibited an opaque green color [34,35].

### 2.7. Data Analysis

Prior to analysis, the data were subjected to the Shapiro–Wilk and Bartlett tests to verify the assumptions of normality and homogeneity of variances (*p* > 0.05). Next, the data were analyzed using analysis of variance (ANOVA) with the F-test, and the means were compared using Tukey’s test at a 5% significance level. The analyses were performed using SISVAR^®^ software, version 5.6. [36], and the graphs were created in SigmaPlot^®^ version 14.0.

In addition, a Principal Component Analysis (PCA) was performed to explore the relationships among physiological variables, photosynthetic pigments, and yield, as well as to identify those variables that contributed most to distinguishing between treatments. PCA was used as a complementary tool to the univariate analyses, allowing for an integrated interpretation of the plants’ responses to the different treatments. The analyses were performed in the RStudio^®^ environment, version 4.3 [37], using the FactoMineR and Factoextra packages.

## 3. Results

### 3.1. Photosystem II Efficiency

According to Table 2, the initial, maximum, and variable fluorescence values were influenced by the interaction between saline water, calcium sources, and application methods (*p* ≤ 0.05). The saline water × calcium source interaction had a significant effect on PSII quantum efficiency (Fv/Fm).

**Figure 4:**
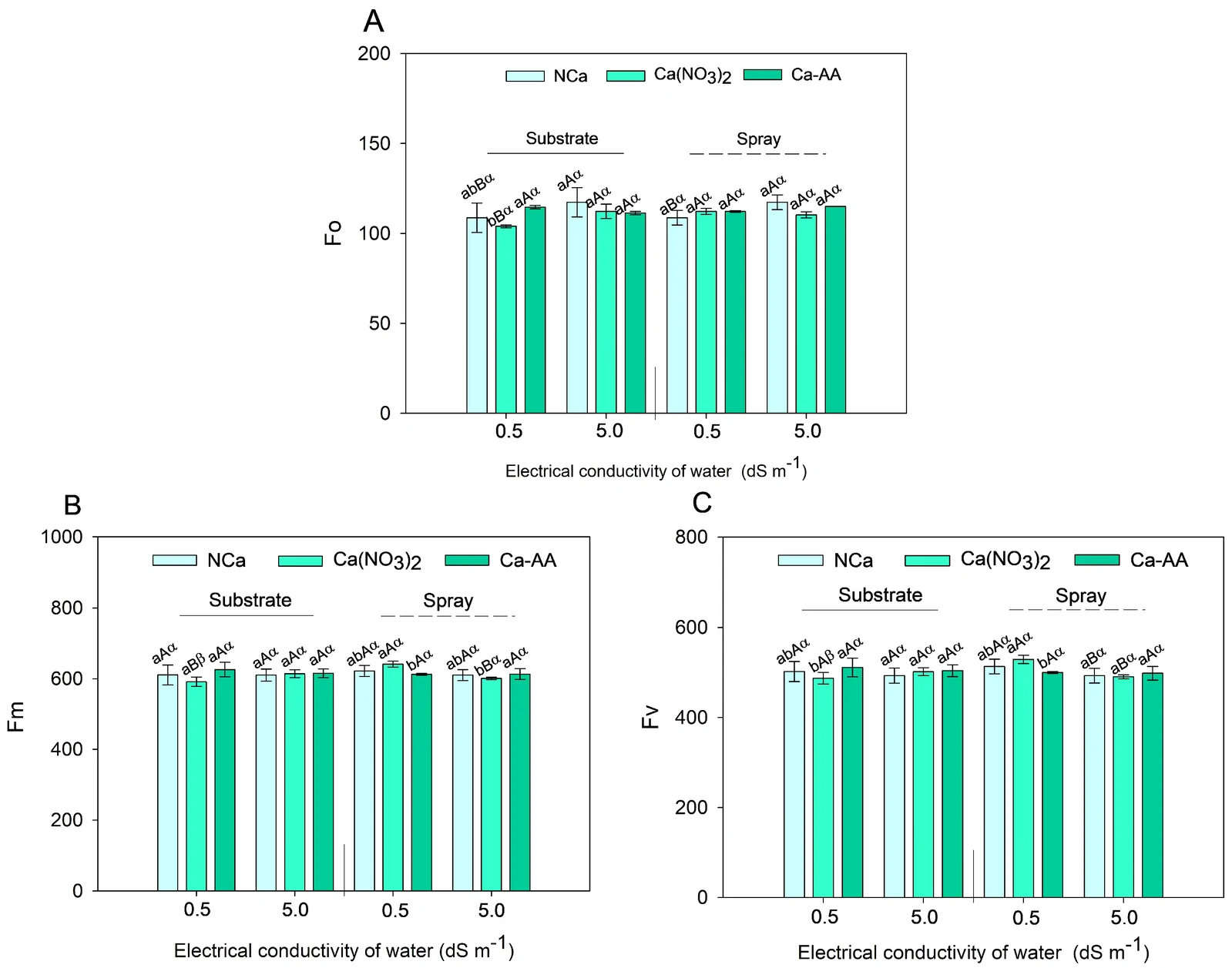
Initial fluorescence (Fo) (**A**), maximum fluorescence (Fm) (**B**), and variable fluorescence (Fv) (**C**) of bell pepper plants under saline irrigation and different methods of calcium application. Bars with the same lowercase letters do not differ from one another for calcium sources within saline solutions and application methods, as determined by the Tukey test (*p* > 0.05). Bars with the same uppercase letters do not differ from one another for saline solutions within calcium sources and application methods, as determined by the F-test (*p* > 0.05). Bars with the same Greek letters do not differ from one another for application methods within calcium sources and saline water, as determined by the F-test (*p* > 0.05). Error bars represent the standard deviation of the biological replicates (n = 4). Statistical differences among treatments were determined by analysis of variance followed by the appropriate mean comparison test.

In both application methods, irrigation with water at 5.0 dS m^−1^ increased the plants initial fluorescence—in the absence of Ca—by 7.31% (Figure 4A), but had no significant effect on maximum fluorescence (Figure 4B). Saline stress did not reduce the Fv of plants without calcium supplied via the substrate, but caused a 4.1% reduction when applied via spray application (Figure 4C).

Under saline irrigation conditions, the calcium sources did not differ significantly for Fo and Fv within each application method (Figure 4A,C), according to the mean comparison test. Although the three-way interaction among saline water, calcium sources, and application methods was significant (Table 2), the effects of calcium sources were dependent on the specific combination of factors, and no differences among calcium sources were detected under the saline conditions evaluated. Meanwhile, in spray application, calcium complexed with amino acids produced the highest Fm values (613.25), whereas no significant differences were observed among the sources when applied via the substrate (Figure 4B). In plants irrigated with non-saline water, substrate application of Ca-AA resulted in the highest values for initial, maximum, and variable fluorescence, whereas, with spray application, calcium nitrate increased the plants’ maximum and variable fluorescence (Figure 4B,C).

The method of application of the calcium sources had no significant effect on initial fluorescence (Figure 4A). In plants irrigated with non-saline water, however, Fv and Fm increased by 8.45% and 8.56%, respectively, when calcium nitrate was applied via spray application.

**Figure 5:**
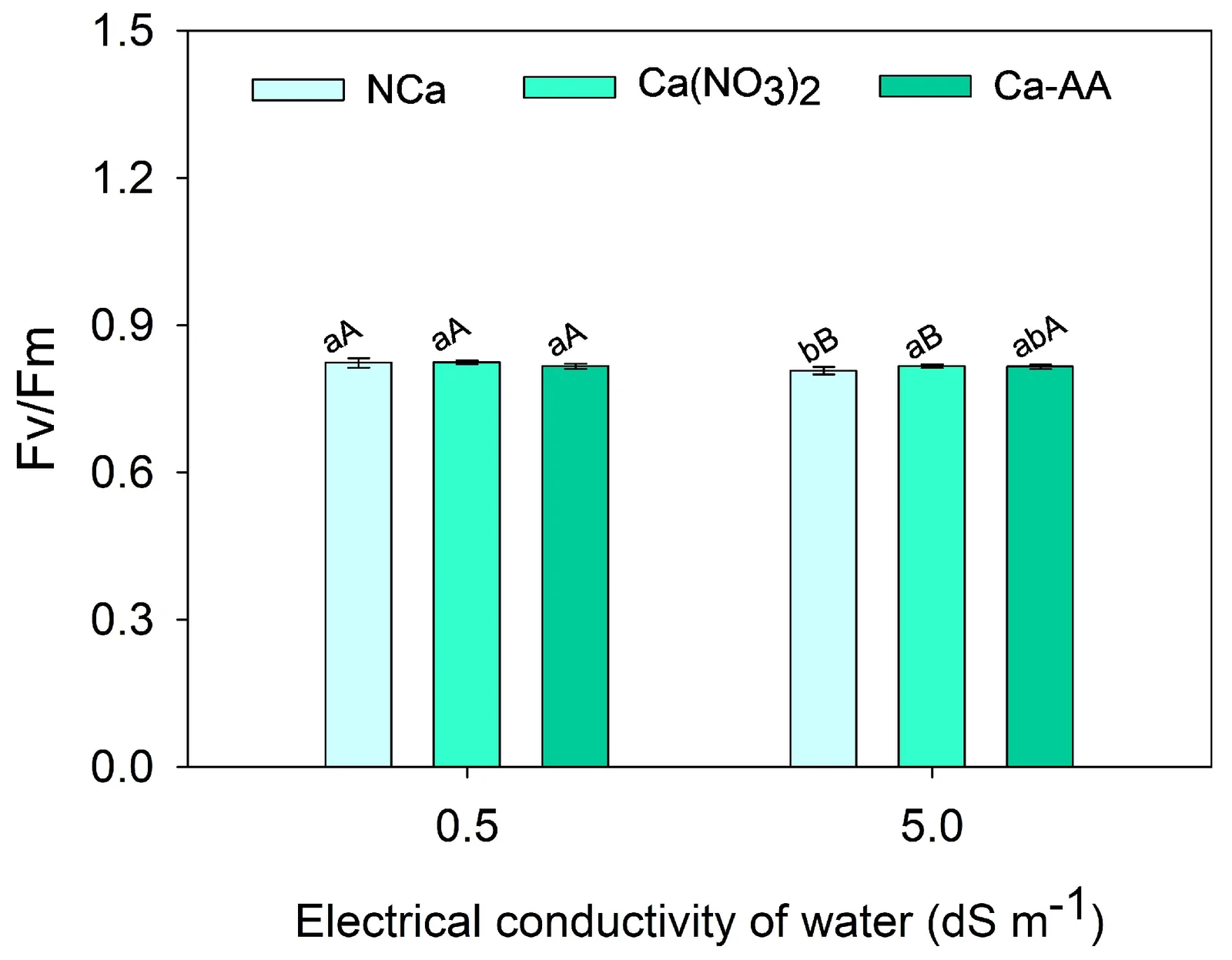
PSII quantum yield—Fv/Fm of bell pepper plants irrigated with saline water and treated with calcium sources under conditions of irrigation water salinity. Bars labeled with the same lowercase letters do not differ from one another for calcium sources within each saline solution, according to the Tukey test (*p* > 0.05). Bars with the same uppercase letters do not differ from one another for saline solutions within each calcium source, as determined by the F-test (*p* > 0.05). Error bars represent the standard deviation of the biological replicates (n = 4). Statistical differences among treatments were determined by analysis of variance followed by the appropriate mean comparison test.

Bell pepper under salt stress reduced the quantum yield from 0.823 to 0.807 compared to plants without stress (Figure 5). Although there was no significant difference among the calcium sources in terms of PSII quantum yield in plants under salt stress, calcium nitrate yielded the highest values for this variable (Figure 5). In plants irrigated with water at 0.5 dS m^−1^, there was no significant difference in Fv/Fm among the calcium sources, with a mean value of 0.821.

### 3.2. Gas Exchange

The AM × SW × CaS interaction had a significant effect on stomatal conductance (*p* ≤ 0.01); the AM × SW interaction had a significant effect on the net CO_2_ assimilation rate and instantaneous carboxylation efficiency (*p* ≤ 0.05), as shown in Table 3. When considered in isolation, water salinity had a significant effect on leaf transpiration and internal CO_2_ concentration (*p* ≤ 0.01). Water use efficiency and intrinsic water use efficiency did not respond to the treatments, with mean values of 4.89 µmol CO_2_ mmol^−1^ H_2_O and 0.053 µmol CO_2_ mol^−1^ H_2_O.

**Figure 6:**
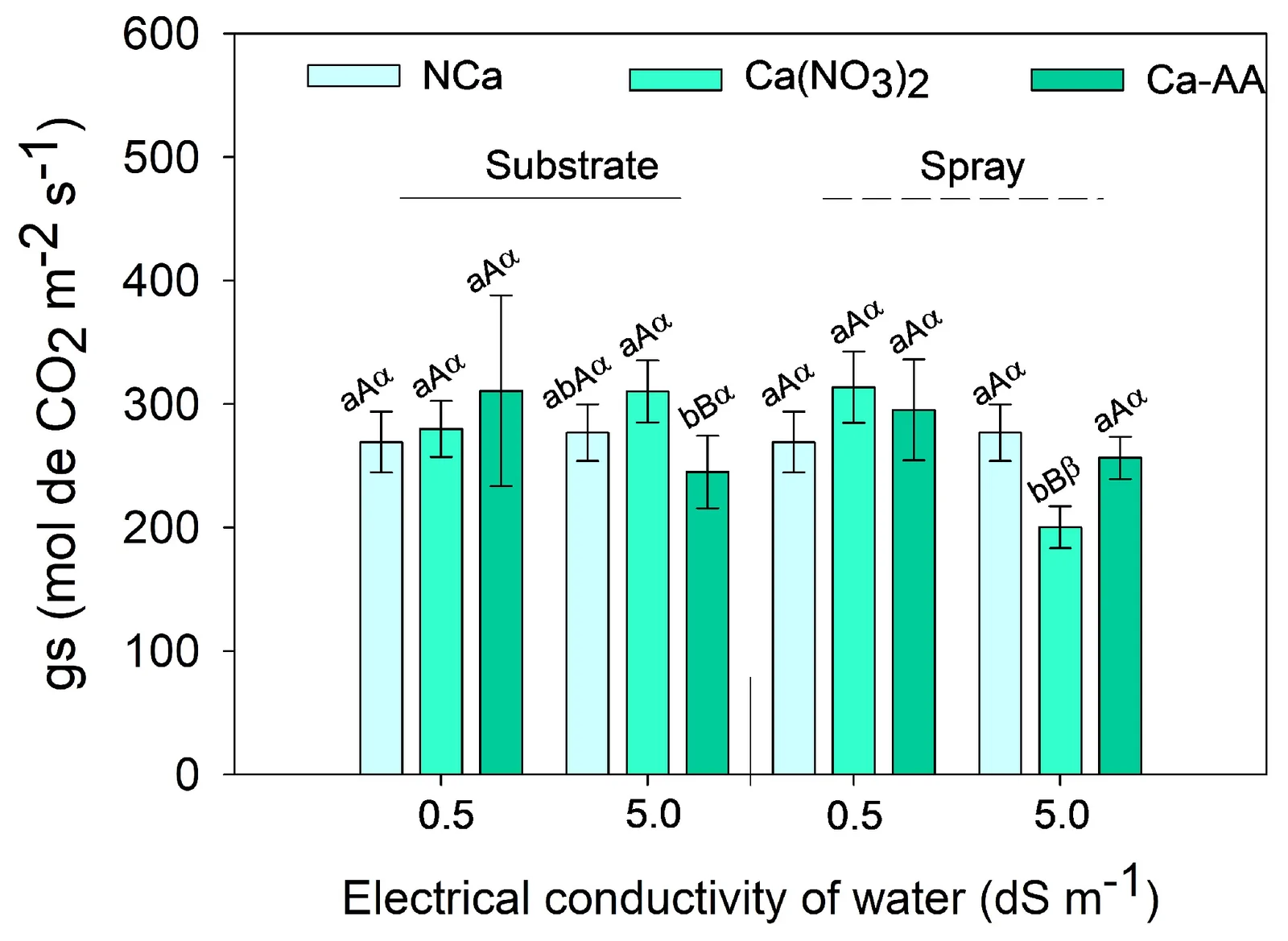
Stomatal conductance—gs of bell peppers under irrigation with saline solution, calcium sources, and application methods. Bars with the same lowercase letters do not differ from one another for calcium sources within each water electrical conductivity level and application method, as determined by the Tukey test (*p* > 0.05). Bars with the same uppercase letters do not differ from one another for saline water within calcium sources and application methods, as determined by the F-test (*p* > 0.05). Bars with the same Greek letters do not differ from one another for application methods within calcium sources and saline water, as determined by the F-test (*p* > 0.05). Error bars represent the standard deviation of the biological replicates (n = 4). Statistical differences among treatments were determined by analysis of variance followed by the appropriate mean comparison test.

As shown in Figure 6, the salinity of the irrigation water did not have a significant effect on the stomatal conductance of bell peppers. However, the application of calcium nitrate via the substrate resulted in higher gs values in plants irrigated with water at 5.0 dS m^−1^, although this did not differ statistically from the treatments without calcium, while the application of Ca-AA reduced stomatal conductance by 11.45% compared to plants without Ca. In contrast, with spray application, the highest gs values in plants irrigated with saline water were observed, respectively, in the treatments without calcium and with calcium complexed with amino acids, which were 38.4% and 28.10% higher, respectively, than those in the calcium nitrate treatment.

In plants irrigated with non-saline water, no significant differences in stomatal conductance were observed when comparing the calcium sources under both application methods (Figure 6). When examining the effect of application methods within the context of water salinity and calcium sources, a significant effect was observed with the application of Ca(NO_3_)_2_ to plants irrigated with water at 5.0 dS m^−1^, with the substrate application being 56.72% higher than the spray application.

**Figure 7:**
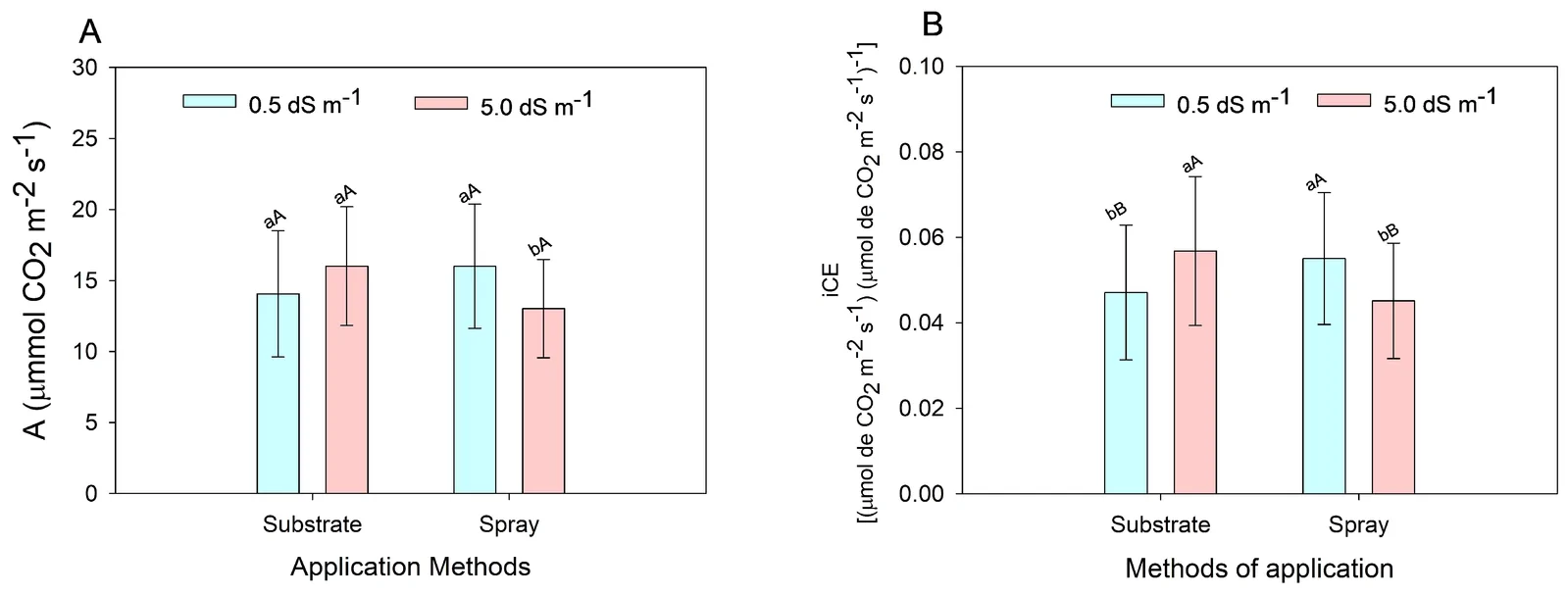
Net CO_2_ assimilation rate—A (**A**) and instantaneous carboxylation efficiency—iCE (**B**) of bell peppers irrigated with saline water and different calcium application methods. Bars with the same lowercase letters do not differ from one another for saline solution according to the F-test (*p* > 0.05). Bars with the same uppercase letters do not differ from one another for the application methods according to the F-test (*p* > 0.05). The bars represent the standard deviation of the sample (n = 4).

According to Figure 7A, it appears that the salinity of the irrigation water did not significantly affect the plants’ net CO_2_ assimilation rate when calcium sources were applied via the substrate. However, salt stress reduced the rate from 16 to 13 µmol m^−2^ s^−1^, representing a loss of 18.75%. The method of calcium application had no significant effect on the plants’ net CO_2_ assimilation rate.

When calcium sources were supplied via the substrate, the plants exhibited higher iCE values under saline conditions, with increases of 20.6% (Figure 7B). In contrast, plants that received calcium sources via spray application exhibited higher iCE values when irrigated with non-saline water (21.95%).

**Figure 8:**
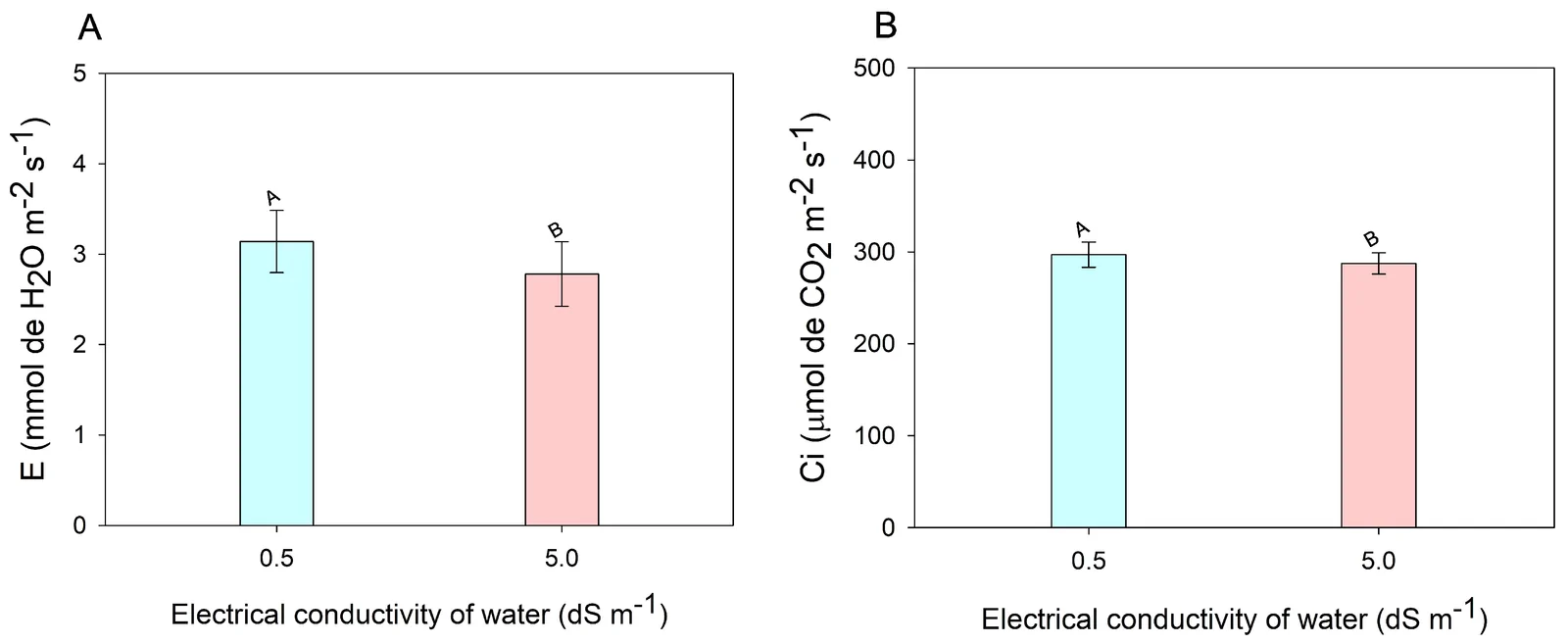
Foliar transpiration—E (**A**) and internal CO_2_ concentration—(Ci) (**B**) in bell peppers irrigated with saline water. Bars with the same letters do not differ from one another according to the F-test (*p* > 0.05). Error bars represent the standard deviation of the biological replicates (n = 4). Statistical differences among treatments were determined by analysis of variance followed by the appropriate mean comparison test.

Saline stress in the water significantly reduced leaf transpiration and internal CO_2_ concentration in bell peppers, with decreases of 11.43% and 3.23%, respectively (Figure 8A,B).

### 3.3. Chlorophyll Indices and Fruit Production

According to the ANOVA (Table 4), the SW × CaS × AM interaction had an effect only on bell pepper yield (*p* ≤ 0.01). The combination of calcium sources and application methods had a significant effect on the leaf chlorophyll *a* and *b* indices (*p* ≤ 0.05), while the *total* chlorophyll index responded to the sources of variation independently for saline water (*p* ≤ 0.05) and calcium sources (*p* ≤ 0.01).

**Figure 9:**
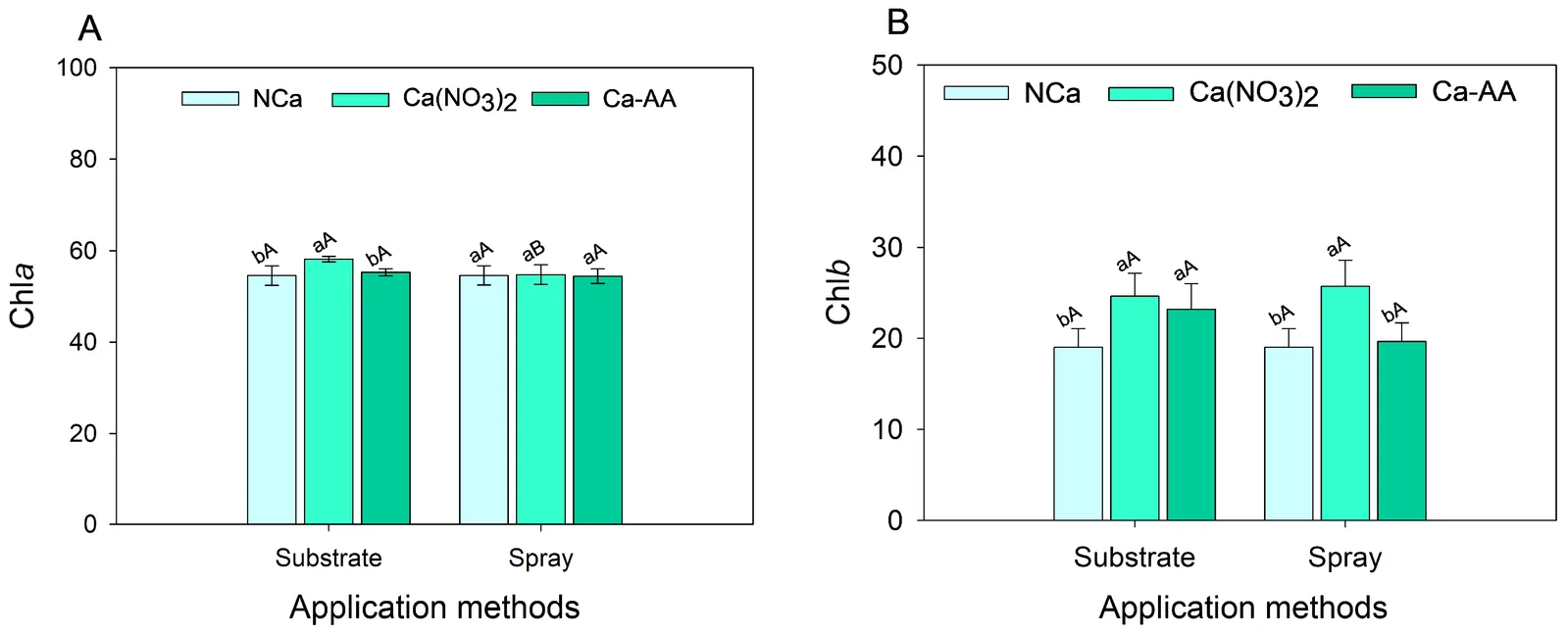
Chlorophyll a—Chl*a* (**A**) and chlorophyll b—Chl*b* (**B**) indices of bell peppers irrigated with saline water and different methods of calcium application. Bars with the same lowercase letters do not differ from one another for calcium sources within each application method, as determined by the Tukey test (*p* > 0.05). Bars with the same uppercase letters do not differ from one another for application forms for each calcium source, as determined by the F-test (*p* > 0.05). Error bars represent the standard deviation of the biological replicates (n = 4). Statistical differences among treatments were determined by analysis of variance followed by the appropriate mean comparison test.

When analyzing the effect of different calcium sources on chlorophyll a level within each application method, for substrate application, it was found that calcium nitrate resulted in the greatest increases—6.51% and 5.98%, respectively—compared to treatments without calcium and with complexed calcium (Figure 9A). Meanwhile, in spray application, no statistically significant difference was observed among the calcium sources, with a mean value of 54.59. Regarding application methods, substrate application was more effective in increasing the plants’ foliar Chl*a* than spray application, with an increase of 6.14%.

Via the substrate, the calcium sources increased the chlorophyll b index values by 29.62% with Ca(NO_3_)_2_ and by 22.11% with Ca-AA, compared to the treatments without calcium (Figure 9B). When applied as a spray, calcium nitrate yielded the highest chlorophyll b index (ICb) values, with a 35.35% increase compared to treatments without calcium and calcium complexed with amino acids. Based on the means, no significant difference was observed between the application methods within each calcium source for ICb.

**Figure 10:**
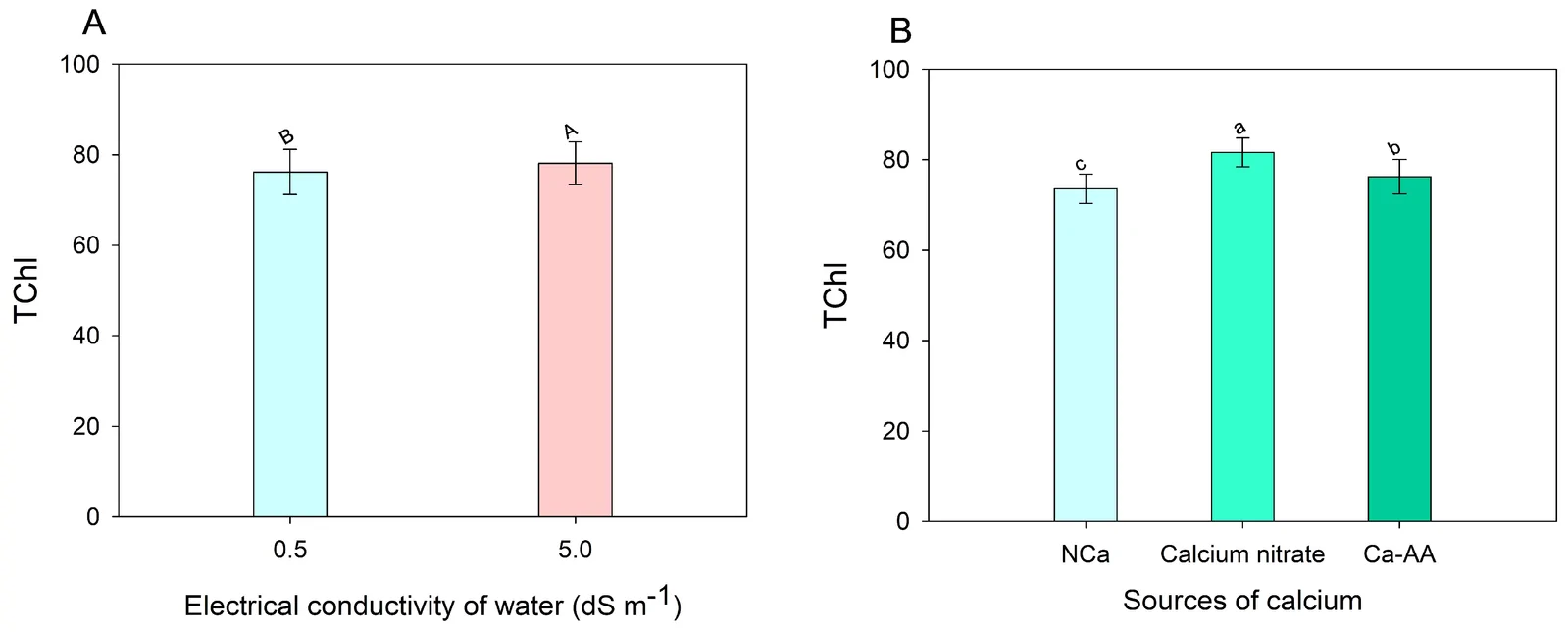
Total chlorophyll index—TChl of bell peppers irrigated with saline water (**A**) and treated with calcium sources (**B**). Bars with the same uppercase letters do not differ from one another for saline solution according to the F-test (*p* > 0.05), and bars with the same lowercase letters do not differ from one another for calcium sources according to Tukey’s test (*p* > 0.05). Error bars represent the standard deviation of the biological replicates (n = 4). Statistical differences among treatments were determined by analysis of variance followed by the appropriate mean comparison test.

The total chlorophyll index of bell pepper leaves showed a statistically significant difference among the irrigation waters and increased from 76.20 to 78.11 as water salinity rose from 0.5 to 5.0 dS m^−1^ (Figure 10A). According to Figure 10B, the application of calcium sources increased the plants’ TChl, particularly calcium nitrate, which showed a 10.93% increase compared to plants in the treatment without calcium.

**Figure 11:**
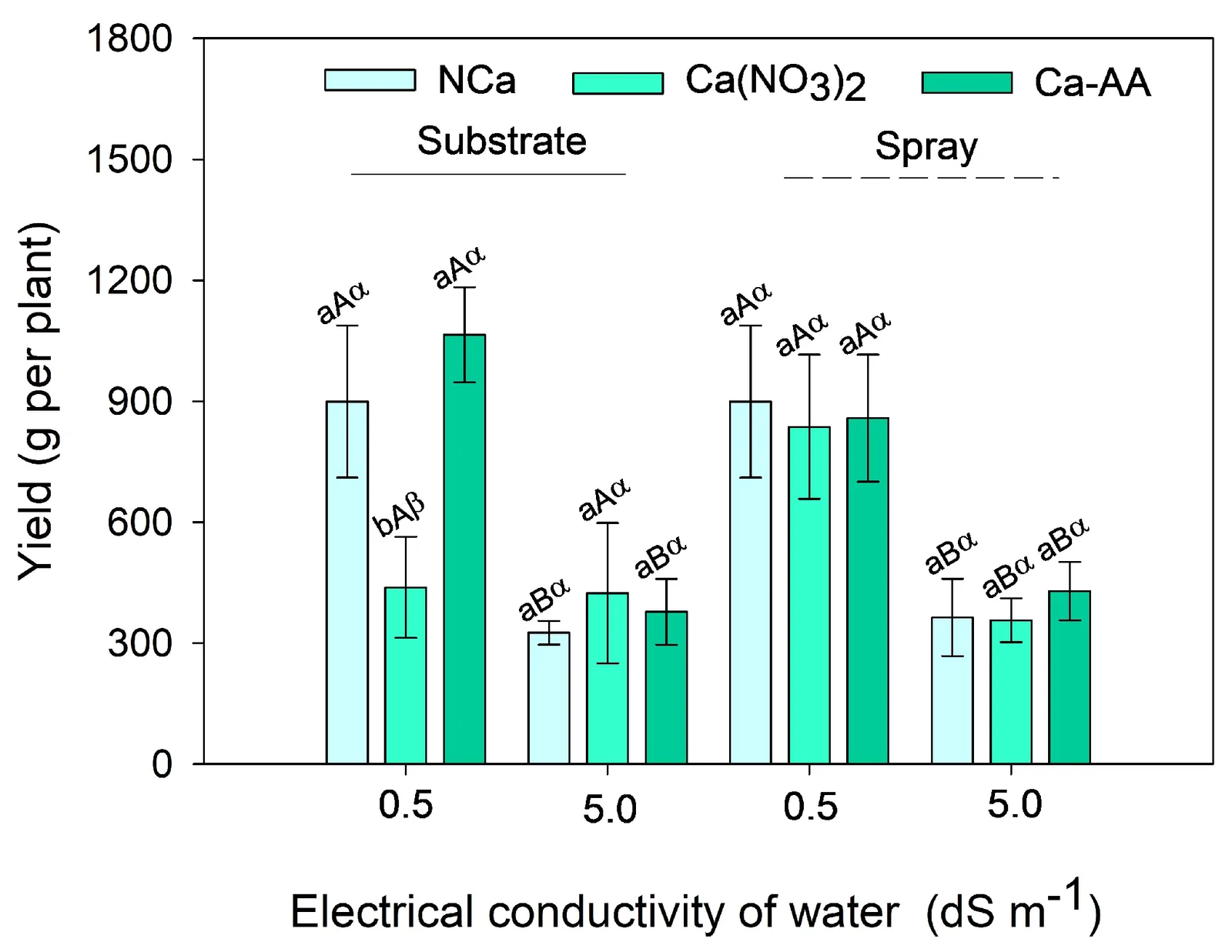
Bell pepper production under saline irrigation and methods of applying calcium sources. Bars with the same lowercase letters do not differ from one another for calcium sources within saline solutions and application methods, as determined by the Tukey test (*p* > 0.05). Bars with the same uppercase letters do not differ from one another for saline solutions within calcium sources and application methods, as determined by the F-test (*p* > 0.05). Bars with the same Greek letters do not differ from one another for application methods within calcium sources and saline water, as determined by the F-test (*p* > 0.05). Error bars represent the standard deviation of the biological replicates (n = 4). Statistical differences among treatments were determined by analysis of variance followed by the appropriate mean comparison test.

As shown in Figure 11, saline stress in the water reduced per-plant yield of bell peppers, with losses of 63.77% in the substrate treatment and 59.58% in the spray treatment. Under saline stress conditions, no effect was observed for the application of calcium sources or for the application methods. In plants irrigated with non-saline water, when calcium sources were applied via the substrate, the highest yield values were observed with the application of calcium chelated with amino acids, at 1065.56 g plant^−1^, but this did not differ significantly from the treatment without calcium and was 143.1% higher than the treatment with calcium nitrate. When calcium sources were applied via spray, no statistically significant difference in fruit yield was observed. Regarding the application method, only in the treatment of plants irrigated with water at 0.5 dS m^−1^ was the spray application of calcium nitrate (836.56 g plant^−1^) more effective than substrate application (438.20 g plant^−1^), with a 90.95% increase in yield.

**Figure 12:**
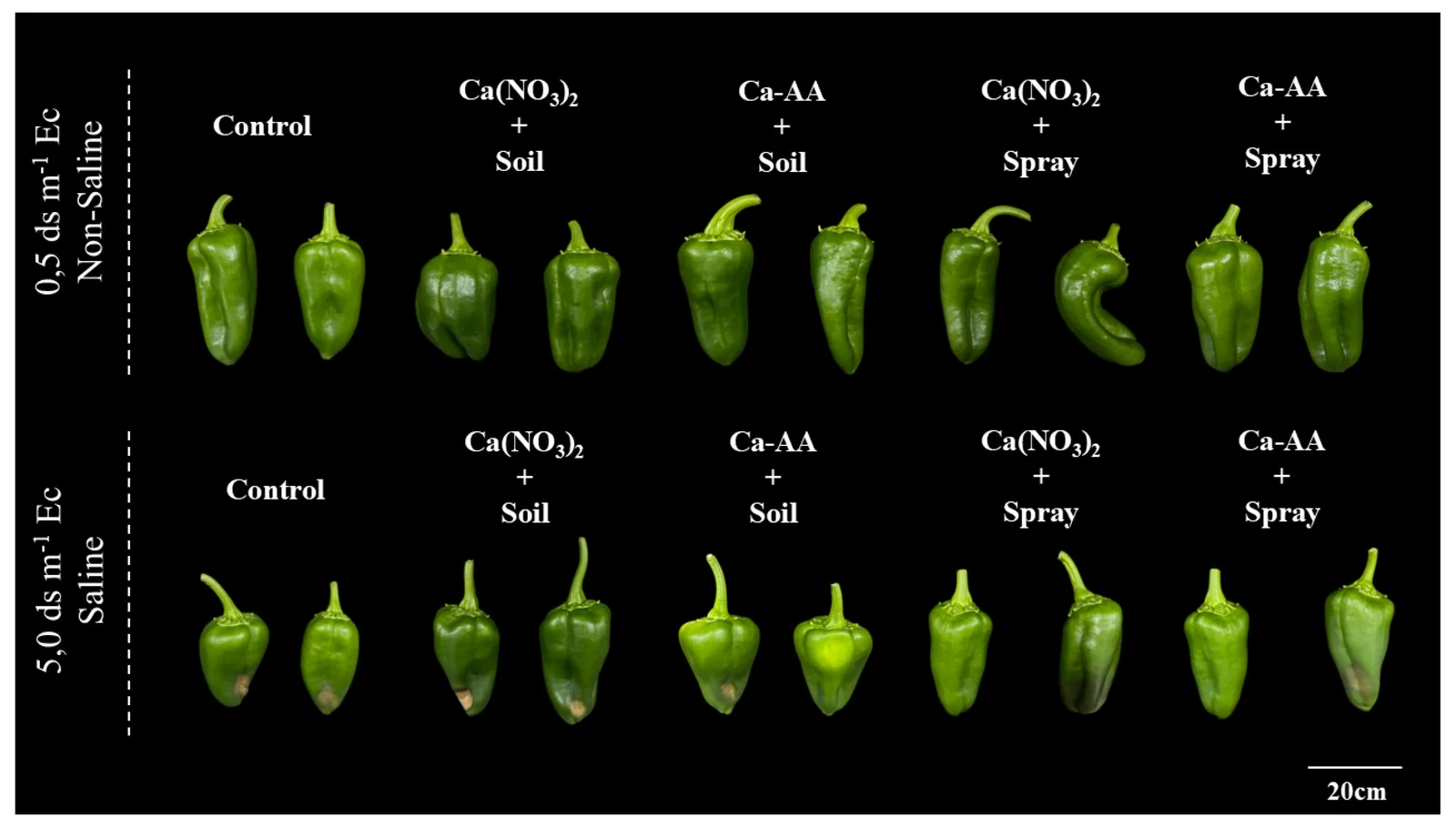
Appearance of bell pepper fruits grown under two electrical conductivities of irrigation water (EC: 0.5 and 5.0 dS m^−1^), calcium sources [no calcium—NCa, calcium nitrate, Ca(NO_3_)_2_, and calcium complexed with amino acids (Ca-AA)], and two application methods (substrate and spray). The images show morphological differences and variations in fruit development in response to the treatments. Scale bar: 20 cm.

The effects of salt stress became visually evident in the growth of bell pepper fruits when the plants were irrigated with water at 5.0 dS m^−1^, with a reduction in fruit size and more pronounced morphological changes in the fruits for the treatments without calcium (Figure 12). Supplementation with amino acid-chelated calcium contributed to greater uniformity and structural preservation, as well as a reduction in fruits exhibiting apical rot—a symptom characteristic of calcium deficiency in bell peppers—thereby reinforcing the mitigating effect of this calcium source under salinity conditions with water at 5.0 dS m^−1^.

In general, the highest production values occurred under lower salinity (0.5 dS m^−1^), particularly for calcium complexed via the substrate, while the lowest values were recorded under high salinity (5.0 dS m^−1^), especially in the treatments without calcium and those with spray calcium nitrate.

### 3.4. Principal Component Analysis (PCA)

**Figure 13:**
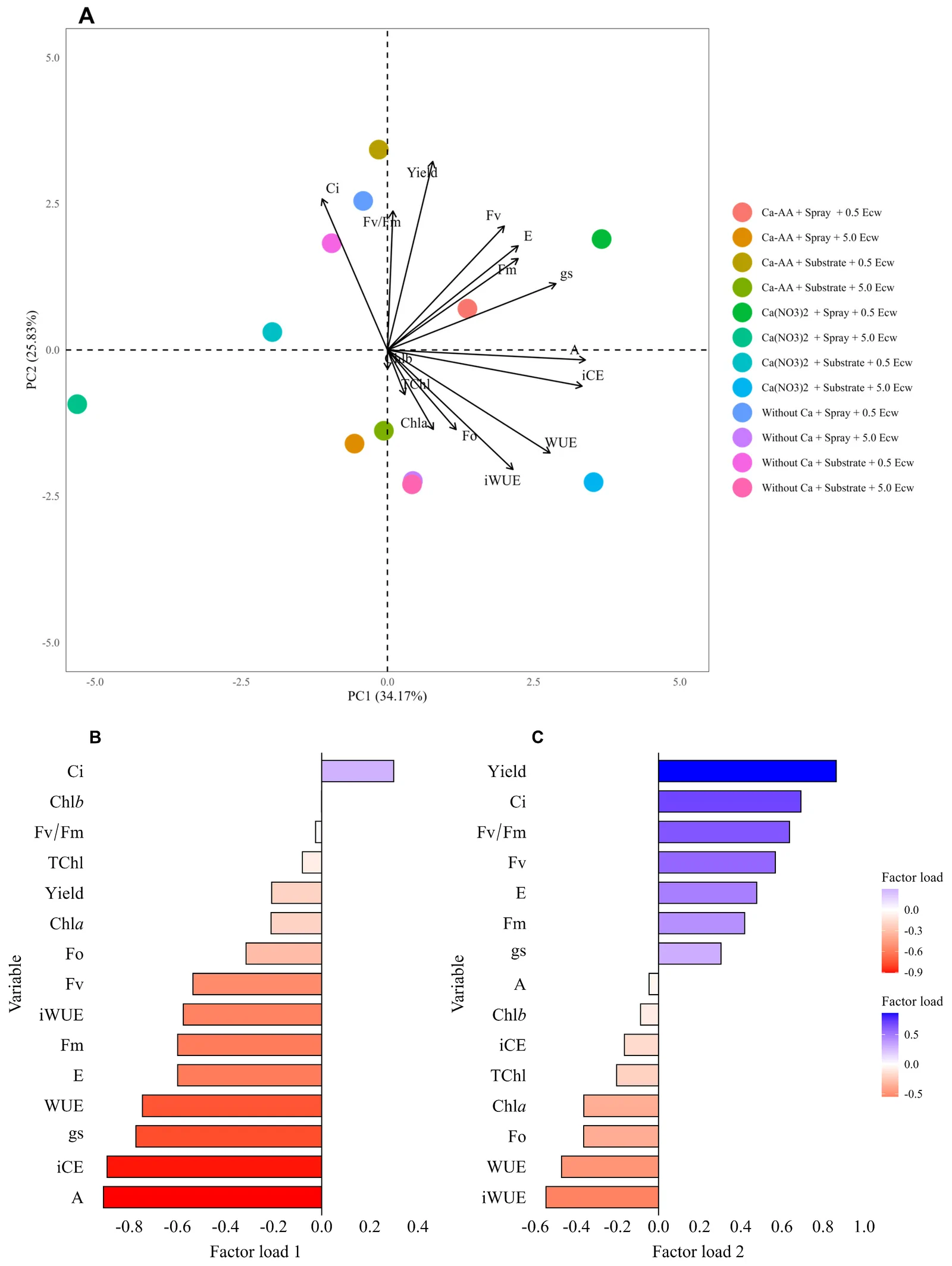
Principal component analysis (PCA) of the treatments (**A**) and variables analyzed (**B**,**C**). ECiw: electrical conductivity of irrigation water; Fo: initial fluorescence; Fm: maximum fluorescence; Fv: variable fluorescence; Fv/Fm: PSII quantum efficiency; gs: stomatal conductance; A: net CO_2_ assimilation rate; Ci: intercellular CO_2_ concentration; E: transpiration rate; iCE: instantaneous carboxylation efficiency; WUE: water use efficiency; iWUE: intrinsic water use efficiency. Chl*a*: Chlorophyll a index; Chl*b*: Chlorophyll b index; TChl: Total chlorophyll index; Yield: Yield.

In the principal component analysis (PCA), the first two principal components jointly explained 60.0% of the total variance, with PC1 and PC2 accounting for 34.17% and 25.83%, respectively, indicating an adequate multivariate representation of the physiological responses (Figure 13A).

PC1 was primarily defined by variables associated with carbon assimilation and photosynthetic performance, exhibiting high factor loadings for A (−0.907), iCE (−0.892), gs (−0.772), WUE (−0.745), E (−0.599), Fm (−0.599), iWUE (−0.576), and Fv (−0.536) (Figure 13B). These variables were oriented in the same direction along the axis, indicating a strong positive association among them and representing a physiological gradient related to photosynthetic activity. In contrast, Ci showed the highest positive loading (0.299), indicating an opposite pattern relative to the variables associated with carbon assimilation and stomatal conductance.

PC2 explained 25.83% of the total variance and was characterized by strong positive contributions from Yield (0.864), Ci (0.693), Fv/Fm (0.637), Fv (0.568), E (0.478), Fm (0.419), and gs (0.304), whereas iWUE (−0.548), WUE (−0.472), Fo (−0.365), and Chl*a* (−0.364) exhibited the highest negative loadings (Figure 13C). This component distinguished variables related to productive performance and photochemical efficiency from those associated with water-use efficiency and basal chlorophyll fluorescence.

The PCA biplot (Figure 13A) revealed a clear separation of treatments according to calcium source, application method, and salinity level. Treatments located in the positive region of PC2 were more closely associated with Yield, Fv/Fm, Fv, Fm, E, and gs, whereas those positioned in the negative region were more strongly associated with WUE, iWUE, Fo, and Chl*a*. Along PC1, treatments distributed on the negative side of the axis were primarily associated with A, iCE, gs, WUE, E, Fm, iWUE, and Fv, whereas those located on the positive side were more closely associated with Ci, indicating a gradient related to carbon assimilation and stomatal functioning.

Overall, both calcium sources were associated with favorable physiological responses under saline conditions. However, treatments receiving Ca-AA clustered more closely with variables related to gas exchange and photosynthetic performance, whereas treatments supplied with Ca(NO_3_)_2_ were primarily associated with yield and photochemical efficiency. These findings indicate that both calcium sources contributed to mitigating the adverse effects of salinity, although Ca-AA exhibited a stronger multivariate association with the photosynthetic and physiological performance of pepper plants.

## 4. Discussion

In agricultural settings, plants are often exposed to various abiotic stresses, which impair physiological and metabolic processes essential for plant growth, development, and yield [38,39]. Under these conditions, plants activate acclimation mechanisms aimed at minimizing cellular damage and restoring physiological homeostasis, particularly through adjustments related to gas exchange and the functioning of the photosynthetic apparatus, including photosynthesis, transpiration, stomatal conductance, and the photochemical efficiency of the photosystems [40,41]. Changes in these mechanisms directly affect the utilization of light energy, CO_2_ assimilation, and the production of photoassimilates, negatively impacting crop growth and yield [38,39,42].

Plants subjected to salt stress exhibit an imbalance between the light energy absorbed and its utilization in photochemical processes, resulting from limited CO_2_ assimilation, which reduces the demand for ATP and NADPH generated during the light phase [41,43]. In this scenario, stomatal closure and metabolic constraints on carbon fixation exacerbate this imbalance, leading to the accumulation of excited energy in photosystem II and increasing the risk of photoinhibition and photooxidative damage to the photosynthetic apparatus [44]. As a result, there is an increase in the production of reactive oxygen species, raising the risk of photoinhibition and damage to the photosynthetic apparatus [41,45,46].

Excess free energy increases the stress on the photosynthetic apparatus, resulting in a reduction in maximum photochemical efficiency, as evidenced by a decrease in the Fv/Fm ratio [10,11]. These changes are associated with structural and functional alterations in PSII, including damage to the D1 protein, whose proteolytic degradation and subsequent replacement by a new copy form the basis of the photosystem II repair cycle [47] and disorganization of the thylakoid membranes, as evidenced by changes in the ultrastructure of the granal thylakoids [48] and in the migration dynamics of PSII complexes between membrane domains [49].

Evidence suggests that salinity reduces the photochemical efficiency of photosystem II and impairs electron transport, contributing to a decrease in the Fv/Fm ratio and changes in the efficiency of the reaction centers [12]. In this study, salt stress led to a reduction in photochemical efficiency, indicating a limitation in the use of light energy and possible partial inactivation of PSII reaction centers. Consequently, an increase in heat dissipation was observed as a photoprotective mechanism, often associated with the activation of the xanthophyll cycle, which acts to dissipate excess energy under stress conditions [44,50,51]. This energy redirection is directly related to photoinhibition and increased non-photochemical dissipation [52].

The fluorescence results generally show that salinity caused changes in photochemical efficiency, with reductions in Fv/Fm, indicating impairment of PSII (Figure 5). This saline response disrupts the organization of thylakoid protein complexes and photosynthetic supercomplexes, reducing the efficiency of light capture, energy transfer, and electron transport in the photosystems [53,54,55].

For the maximum quantum yield of PSII (Fv/Fm), values close to 0.82 under low-salinity conditions indicate that the photosynthetic apparatus is functioning at full capacity [56]. However, under high-salinity conditions, the 2.44% reduction observed in plants without calcium supplementation indicates the occurrence of photoinhibition, albeit at a relatively small level. On the other hand, the maintenance of higher values in plants treated with Ca(NO_3_)_2_ and CA-AA reinforces the role of calcium in protecting PSII. According to Vafadar and Ehsanzadeh [57], spray application of calcium (5 mM) increased the maximum primary photochemical quantum yield of PSII in *Dracocephalum kotschyi* plants subjected to 100 mM NaCl salinity, acting as a cellular signal and mitigating the effects of salt stress. Ca^2+^ acts directly to stabilize thylakoid membranes and maintain the activity of reaction centers, promoting the recovery of electron transport and reducing photo-oxidative damage [21].

The addition of calcium promoted a partial recovery of the parameters, corroborating its structural role in the oxygen-evolving complex (OEC). Ca^2+^ contributes to the stabilization of the Mn_4_CaO_5_ complex, which is responsible for the photolysis of water and the supply of electrons to the photosystem II (PSII) electron transport chain. Excess Na^+^ compromises the integrity of thylakoid membranes and destabilizes PSII-associated proteins, reducing the efficiency of electron transfer and promoting photo-oxidative damage. Furthermore, calcium helps maintain the structural integrity of PSII and reduce lipid peroxidation in thylakoid membranes [8,58].

In addition to calcium itself, the accompanying ions supplied by each fertilizer may also have contributed to the observed physiological responses. Calcium nitrate provides nitrate (NO_3_^−^), an oxidized nitrogen source that requires metabolic reduction before assimilation, thereby consuming reducing power (NADH/NADPH) [59]. In contrast, amino acid-complexed calcium supplies nitrogen in a more reduced form, which may be more readily incorporated into plant metabolism while simultaneously providing amino acids that contribute to osmotic adjustment, metabolic regulation, and stress signaling [60]. Therefore, the contrasting physiological responses observed between calcium nitrate and amino acid-complexed calcium may reflect not only differences in calcium delivery but also the distinct metabolic effects associated with their accompanying nitrogen forms.

Given that the chelated calcium used in this study contains amino acids such as proline, glycine, and glutamic acid in its amino acid profile, the beneficial effects observed on photochemical parameters may be related to the physiological properties attributed to these compounds under saline stress conditions. These amino acids have been associated with osmoregulation, the stabilization of proteins and cell membranes, and the modulation of redox metabolism, contributing to the maintenance of cellular homeostasis [61,62]. In addition, the complexation of Ca^2+^ with amino acids may enhance its bioavailability and efficiency of use by plants [63,64]. However, given that these compounds were not quantified in the plants evaluated, it is suggested that their effects be interpreted as potential physiological contributions associated with the composition of the chelated calcium used.

Amino acids can contribute to the supply of carbon and nitrogen to plants, promoting the synthesis of essential nitrogen-containing compounds, such as chlorophylls, proteins, and enzymes involved in photosynthetic metabolism [63,65]. In addition, amino acids such as glutamate play a role in the formation of chlorophyll precursors and help maintain photosynthetic and antioxidant efficiency under saline stress [66,67,68].

The results of this study show that calcium sources were the main factor determining chlorophyll indices. Calcium nitrate promoted the greatest increases, possibly due to the higher availability of nitrogen contained in this source, since NO_3_^-^ acts directly as a nitrogen source for the formation of the porphyrin ring—the central structure of the chlorophyll molecule—and also modulates gene expression under saline stress conditions, promoting the maintenance of pigments [65,69,70], which helps preserve the pigments even under saline stress.

The approximately 2.5% increase in the total chlorophyll index at 5.0 dS m^−1^ indicates an increase in pigment concentration in plants subjected to stress. Under saline conditions, the increase in chlorophyll indices may be associated with the pigment concentration effect, resulting from reduced biomass production. This behavior is consistent with Ma et al. [71], who observed increases in chlorophyll content associated with a reduction in leaf area and aboveground biomass in cotton, indicating a concentration effect. Additionally, the increase was predominantly associated with chlorophyll a, which contributed most to the total content (a + b), while chlorophyll b accounted for a smaller proportion, in accordance with [72], which also describe this behavior as characteristic of salt-tolerant plants.

In addition, Hand et al. [73] observed in bell peppers (*Capsicum annuum* L.) that tolerant cultivars maintain high chlorophyll levels due to increased activity of antioxidant enzymes (SOD and POD) and the accumulation of osmolytes, which protect the protein-pigment–lipid complex from degradation by chlorophyllase. In the literature, this effect has been associated with the action of these enzymes in chloroplasts, where higher SOD and POD activities may contribute to maintaining the redox state and the integrity of photosynthetic processes under salt stress [74].

Calcium contributes to the integrity of thylakoid membranes and to the stabilization of proteins involved in photosynthesis. An adequate supply of this element has been linked to a reduction in the degradation of chlorophyll molecules and damage to photosynthetic antenna protein complexes, helping to maintain chloroplast structure even under stress [21]. In addition, the presence of Ca^2+^ modulates the reduction in ROS production, exerting a protective effect on the photosynthetic apparatus that promotes an increase in chlorophyll levels [19,75]. This set of effects may help explain the greater increases observed with the use of calcium nitrate in this study.

Despite the maintenance or increase in photosynthetic pigments, as evidenced by the rise in the total chlorophyll index, salinity stress caused changes in the photochemical system, characterized by an increase in initial fluorescence and non-photochemical energy dissipation, as well as a reduction in quantum yield and electron transport efficiency in PSII. As a result, there was a reduction in the photosynthetic rate, leaf transpiration, and internal CO_2_ concentration, reflecting lower carbon assimilation and, consequently, reduced yield per bell pepper plant under salinity stress (63.77% via the substrate and 59.58% via the leaves), confirming the crop’s sensitivity to a salinity of 5.0 dS m^−1^, the reported tolerance limit for this crop [76].

This physiological response can be explained by the fact that the reduction in production under salinity stress is associated with a decrease in soil osmotic potential, which induces stomatal closure, limiting CO_2_ uptake and reducing carboxylation and photosynthetic rates [77]. In addition, the decrease in photochemical efficiency, which compromises PSII, exacerbates photosynthetic limitations, resulting in lower yields. Consequently, plant growth and development are impaired, leading to lower fruit production [78]. A smaller stomatal aperture exacerbates photosynthetic limitations, contributing to reduced bell pepper productivity under saline conditions [79]. Under non-saline water conditions, the higher yield performance of Ca-AA via the substrate suggests that, in the absence of stress, root uptake is not a limiting factor, allowing for greater utilization of calcium complexed with amino acids for plant growth and development.

In a saline environment, Ca(NO_3_)_2_ promoted PSII stability and higher chlorophyll levels; however, its application via the substrate may have intensified the osmotic component of the soil solution due to the additional input of salts, thereby increasing the electrical conductivity of the medium and reducing the agronomic efficiency of fertilization under saline conditions [80]. Furthermore, the assimilation of NO_3_^−^ depends on its sequential reduction to NO_2_^−^ and NH_4_^+^ by the enzymes nitrate reductase and nitrite reductase, processes that can be negatively affected by salinity due to reduced enzyme activity, gene expression, and protein synthesis [81]. Under these conditions, some of the energy and photoassimilates may have been diverted to cellular maintenance and osmotic balance, reducing the flow of carbon to growth and fruit production [23,82].

In contrast, spray application of Ca-AA likely resulted in a lower osmotic impact on the rhizosphere and a greater biostimulant effect, since amino acids can promote photosynthesis by enhancing chlorophyll synthesis and improving electron transfer in PSII, in addition to contributing to protection against oxidative stress by acting directly or indirectly on antioxidant systems [83]. This result is linked to the use of more efficient formulations, since sources containing chelated elements increase the absorption and translocation of the nutrient within plants [27], since chelated compounds are more easily absorbed by roots or leaves than inorganic sources [26].

In the root zone, the use of chelating agents protects calcium from undesirable reactions, such as fixation, precipitation, and leaching, resulting in greater efficiency and better nutrient uptake by plants [84]. As demonstrated by [85] in *Gleditsia sinensis*, supplementation with exogenous Ca^2+^ under saline conditions restores the ionic balance in the roots, increasing Ca^2+^ and K^+^ concentrations and reducing Na^+^ accumulation, which decreases phytotoxicity and helps alleviate salt stress. In addition, amino acids play a fundamental role in plant metabolism, contributing to protein synthesis and essential cellular functions [86]. Glycine, in particular, plays a role in chlorophyll synthesis and in maintaining photosynthetic efficiency, thereby promoting plant growth and yield [87,88,89].

On the other hand, regarding application methods, the greater efficiency of calcium nitrate applied via spray application compared to application via the substrate in terms of increased fruit yield can be explained by the direct supply of Ca^2+^ and N, which allows for a faster physiological response compared to supply via the substrate [90]. Foliar uptake circumvents potential limitations of the root system, particularly those related to calcium mobility in the soil, thereby helping to maintain photochemical activity and carbon assimilation [91], with positive effects on bell pepper fruit production.

Supporting this finding, the greater efficiency of spray calcium application can be understood by considering that Ca^2+^ is transported predominantly via the xylem, following the transpiration flux, and depends on the concentration of calcium in the xylem sap and on factors such as the microclimate of the canopy [92,93]. In this regard, Ca^2+^ is considered to have low mobility in the phloem, requiring a continuous supply to meet growth demands, especially in leaves and roots [94].

Under salinity stress, reductions in the photosynthetic rate, transpiration, stomatal conductance, and water uptake by the roots impair the transport and uptake of Ca^2+^ in the leaves and roots [15,95]. Given this, foliar fertilization has been used to supplement root fertilization, especially to supply nutrients with low mobility, such as Ca^2+^ [84,96]. Supplementation with exogenous Ca^2+^ can reverse the negative effects of salinity by modulating the physiological and biochemical characteristics of plants [15]. Thus, the restrictions on Ca^2+^ transport imposed by salt stress may help explain the greater efficiency of spray application observed in this study.

**Figure 14:**
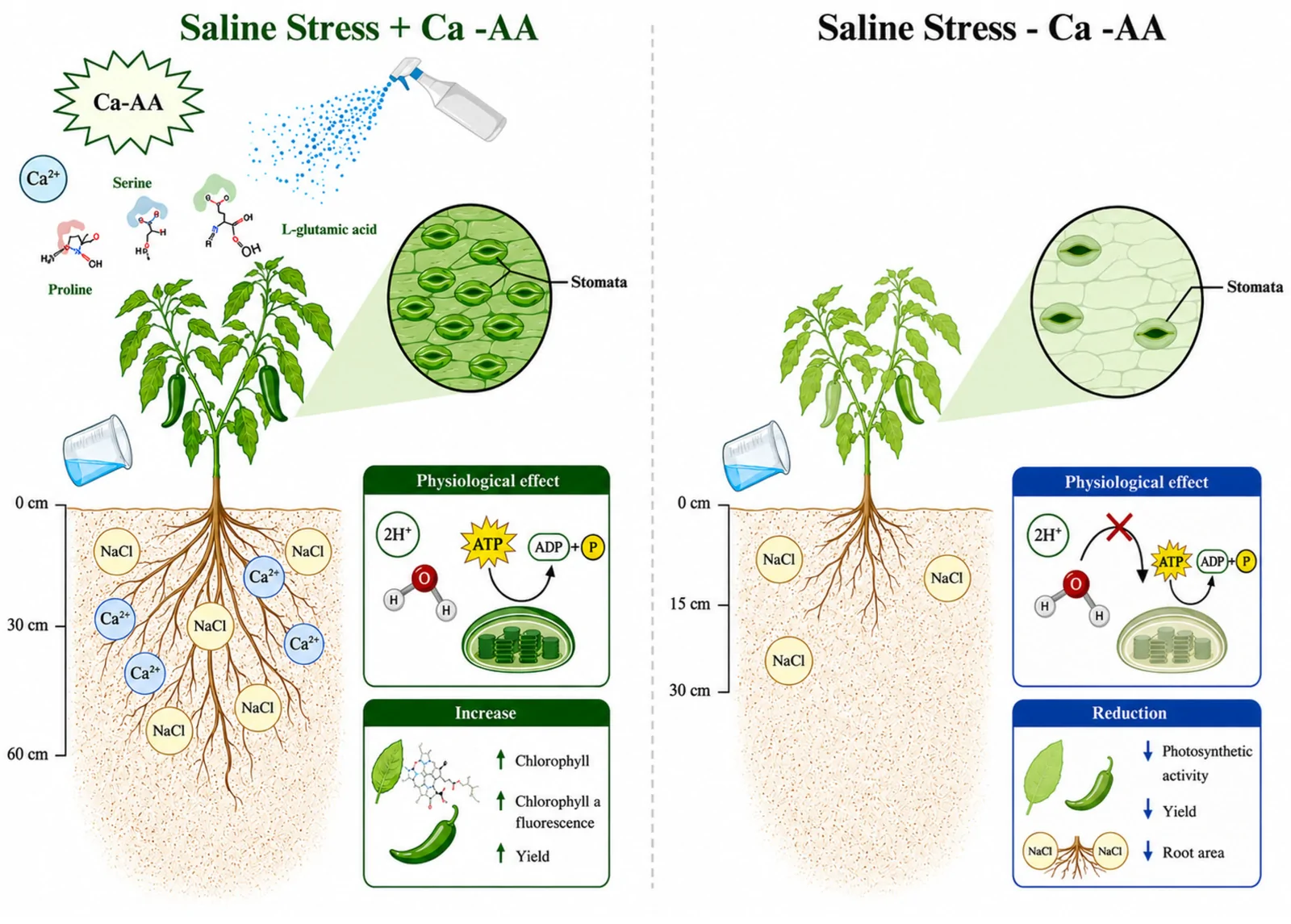
Overview of the effects of amino acid-complexed calcium (Ca-AA) on the mitigation of salinity stress in bell peppers cv. “Cascadura Ikeda”. Figure created with BioRender.com.

The main effects of chelated calcium on bell pepper under salt stress are summarized in the summary graph (Figure 14).

Overall, the reduction in production under salinity stress was associated with lower photochemical efficiency, limitations in gas exchange, and changes in chlorophyll content, compromising carbon assimilation (Figure 14). The application of calcium mitigated the deleterious effects of salinity by preserving the integrity of PSII and promoting the balance of photosynthetic processes.

Ca(NO_3_)_2_ applied via the substrate promoted greater preservation of the photosynthetic apparatus, as reflected in higher fluorescence and chlorophyll values, which likely indicate greater stability of the thylakoid membranes (Figure 14). In contrast, Ca-AA applied via foliar spray showed a better response in production variables, possibly due to the osmoprotective and biostimulant effects of amino acids, which favored osmotic adjustment and the directed transport of photoassimilates to the fruits.

Physiologically, Ca(NO_3_)_2_ applied as a substrate acted primarily through the apoplastic pathway, reducing ionic toxicity and stabilizing cell membranes, while spray-applied Ca-AA may have promoted metabolic responses via the symplastic pathway, contributing to greater osmotic balance and sustaining growth under saline conditions.

## 5. Conclusions

Water salinity reduced gas exchange, chlorophyll content, and photochemical efficiency, impairing carbon assimilation and plant productivity. However, the application of calcium mitigated the effects of salt stress on the physiological and productive performance of bell peppers.

Ca(NO_3_)_2_ applied via the substrate was more effective in preserving the photosynthetic apparatus, resulting in higher chlorophyll content and chlorophyll fluorescence, indicating greater stability of photosystem II. Calcium complexed with amino acids (Ca-AA) applied via spray application led to higher yield under saline conditions.

Future studies should investigate the biochemical and molecular mechanisms involved in the plant response to the combined application of calcium nitrate via the substrate and of calcium complexed with amino acids via spray application, with an emphasis on antioxidant activity, osmotic metabolism, and the expression of genes related to Ca^2+^ uptake, transport, and signaling, with the aim of elucidating the processes responsible for mitigating salt stress in bell peppers and other crops of agronomic interest.

## Figures and Tables

**Table 1:** Water volume per plant at each phenological stage and throughout the growth cycle during the experiment with the bell pepper cv. Cascadura Ikeda.

Phenological Stages	DAT	Volumes of Water Applied (L)
Transplant–Establishment	0–5	9.12
Establishment–Vegetative growth	5–12	13.80
Vegetative growth–Flowering	12–25	23.80
Flowering–Pruning	25–35	41.00
Pruning–Fruit development	35–60	102.65
Fruit development–Harvest	60–75	60.62
Total applied during the cycle	0–75	251.00

DAT—Days after the transplant.

**Table 2:** Summary of the analysis of variance for chlorophyll fluorescence variables in bell pepper irrigated with saline water (SW) under calcium sources (CaS) and application methods (AM).

SV	DF	Values-F			
		Fo	F_m_	F_v_	F_v_/F_m_
Blocks	3	4.47 ^ns^	2.08 ^ns^	1.50 ^ns^	0.98 ^ns^
Application methods (AM)	1	3.46 ^ns^	1.01 ^ns^	0.55 ^ns^	0.04 ^ns^
Residue 1	3				
Saline water (SW)	1	10.46 **	3.07 ^ns^	8.20 **	19.23 **
Calcium sources (CaS)	2	4.00 *	0.57 ^ns^	0.23 ^ns^	2.86 ^ns^
AM × SW	1	0.33 ^ns^	7.83 **	7.42 *	1.01 ^ns^
AM × CaS	2	0.67 ^ns^	4.04 *	3.61 *	0.49 ^ns^
SW × CaS	2	4.95 ^ns^	0.40 ^ns^	0.72 ^ns^	5.04 *
AM × SW × CaS	2	4.30 *	8.69 **	5.76 **	0.14 ^ns^
Residue 2	30				
Total	47				
CV_1_		2.08	3.03	3.85	0.86
CV_2_		3.58	2.12	2.49	0.81
Means		112.00	613.91	501.91	0.817

SV: Sources of variation; DF: Degree of freedom; CV_1_: Coefficient of variation for the main plot (application methods); CV_2_: Coefficient of variation for the subplots (saline water and calcium sources); **, *, and ^ns^: Significant at the 1% and 5% levels, and not significant according to the F-test. Fo: Initial fluorescence; Fm: Maximum fluorescence; Fv: Variable fluorescence; Fv/Fm: PSII quantum efficiency.

**Table 3:** Summary of the analysis of variance for gas exchange variables in bell peppers irrigated with saline water (SW) under different calcium sources (CaS) and application methods (AM).

SV	DF	Values-F						
		gs	A	Ci	E	iCE	WUE	iWUE
Blocks	3	2.08 ^ns^	7.96 ^ns^	13.03 *	3.94 ^ns^	7.34 ^ns^	2.43 ^ns^	0.0004 ^ns^
Application methods (AM)	1	1.39 ^ns^	0.31 ^ns^	1.15 ^ns^	3.94 ^ns^	0.36 ^ns^	0.02 ^ns^	0.0001 ^ns^
Residue 1	3							
Saline water (SW)	1	10.05 **	0.25 ^ns^	7.27 *	20.34 **	0.01 ^ns^	2.52 ^ns^	0.0001 ^ns^
Calcium sources (CaS)	2	0.06 ^ns^	0.02 ^ns^	2.52 ^ns^	0.33 ^ns^	0.02 ^ns^	0.64 ^ns^	0.0001 ^ns^
AM × SW	1	4.59 *	5.26 *	2.93 ^ns^	2.28 ^ns^	5.54 *	0.02 ^ns^	0.0003 ^ns^
AM × CaS	2	1.83 ^ns^	0.87 ^ns^	1.03 ^ns^	1.91 ^ns^	0.91 ^ns^	3.52 ^ns^	0.0001 ^ns^
SW × CaS	2	4.16 *	0.70 ^ns^	0.72 ^ns^	2.16 ^ns^	0.48 ^ns^	1.27 ^ns^	0.0001 ^ns^
AM × SW × CaS	2	8.51 **	2.70 ^ns^	0.74 ^ns^	2.62 ^ns^	2.76 ^ns^	2.14 ^ns^	0.0001 ^ns^
Residue 2	30							
Total	47							
CV_1_		12.04	19.09	1.65	12.19	20.51	16.24	13.44
CV_2_		11.44	25.20	4.21	11.12	28.37	21.62	20.30
Means		275.41	14.72	292.25	2.97	0.051	4.89	0.053

SV: Sources of variation; DF: Degree of freedom; CV_1_: Coefficient of variation for the main plot (application methods); CV_2_: Coefficient of variation for the subplots (saline water and calcium sources); **, *, and ^ns^: Significant at the 1% and 5% levels, and not significant according to the F-test. gs: stomatal conductance; A: net CO_2_ assimilation rate; Ci: intercellular CO_2_ concentration; E: transpiration rate; iCE: instantaneous carboxylation efficiency; WUE: water use efficiency; iWUE: intrinsic water use efficiency.

**Table 4:** Summary of the analysis of variance for the variables of chlorophyll index and bell pepper yield in plants irrigated with saline water (SW) under different calcium sources (CaS) and application forms (AM).

FV	GL	Values-F			
		Chl*a*	Chl*b*	TChl	Yield
Blocks	3	2.28 ^ns^	1.93 ^ns^	1.34 ^ns^	4.22 ^ns^
Application methods (AM)	1	6.92 ^ns^	5.58 ^ns^	6.69 ^ns^	0.87 ^ns^
Residue 1	3				
Saline water (SW)	1	0.73 ^ns^	2.91 ^ns^	4.61 *	200.78 **
Calcium sources (CaS)	2	6.06 **	25.05 **	27.75 **	9.49 **
AM × SW	1	0.08 ^ns^	0.05 ^ns^	0.01 ^ns^	0.77 ^ns^
AM × CaS	2	5.77 **	3.53 *	2.14 ^ns^	4.89 *
SW × CaS	2	2.52 ^ns^	0.94 ^ns^	0.16 ^ns^	10.44 **
AM × SW × CaS	2	0.35 ^ns^	0.81 ^ns^	0.75 ^ns^	11.21 **
Residue 2	30				
Total	47				
CV_1_		3.47	6.15	3.83	21.71
CV_2_		2.74	11.62	4.10	18.28
Means		55.31	21.83	77.10	606.27

SV: Sources of variation; DF: Degree of freedom; CV_1_: Coefficient of variation for the main plot (application methods); CV_2_: Coefficient of variation for the subplots (saline water and calcium sources); **, *, and ^ns^: Significant at the 1% and 5% levels, and not significant according to the F-test. Chl*a*: Chlorophyll a index; Chl*b*: Chlorophyll b index; TChl: Total chlorophyll index; Yield: Yield.

## Data Availability

The original contributions presented in this study are included in the article/supplementary material. Further inquiries can be directed to the corresponding author.

## Supplementary Materials

The following supporting information can be downloaded at: https://www.mdpi.com/article/10.3390/plants15172620/s1.
